# The axonal transport velocity of prions is independent of prion formation

**DOI:** 10.1371/journal.ppat.1014456

**Published:** 2026-07-24

**Authors:** Sam M. Koshy, Jay Hrdlicka, Michael G. Nichols, Anthony S. Stender, Jack H. Taylor, Joaquín Castilla, Jason C. Bartz

**Affiliations:** 1 Department of Medical Microbiology and Immunology, School of Medicine, Creighton University, Omaha, Nebraska, United States of America; 2 Department of Physics, College of Arts and Sciences, Creighton University, Omaha, Nebraska, United States of America; 3 Integrated Biomedical Imaging Facility (IBIF), Creighton University, Omaha, Nebraska, United States of America; 4 Biostatistical core facility, Creighton University, Omaha, Nebraska, United States of America; 5 Center for Cooperative Research in Biosciences (CIC BioGUNE), Basque Research and Technology Alliance (BRTA), Derio, Spain; 6 Centro de Investigación Biomédica en Red de Enfermedades infecciosas (CIBERINFEC), Carlos III National Health Institute, Madrid, Spain; 7 IKERBASQUE, Basque Foundation for Science, Bilbao, Spain; 8 Department of Microbiology, Immunology, and Pathology, Prion Research Center, Colorado State University, Fort Collins, Colorado, United States of America; University of Edinburgh, UNITED KINGDOM OF GREAT BRITAIN AND NORTHERN IRELAND

## Abstract

Prion diseases are caused by PrP^Sc^, the self-templating infectious conformation of the normal host-encoded prion protein, PrP^C^. Spread of PrP^Sc^ in the peripheral and central nervous system follows a highly predictable pattern consistent with slow axonal transport along defined neuroanatomical pathways. The rate of PrP^Sc^ transport, the contribution of prion strain diversity, and the requirement for prion conversion during transport are poorly understood, in part, due to a lack of direct measurement of PrP^Sc^ transport under defined conditions. Here, using a combination of live animal and sciatic nerve explant culture experiments, and determined the velocity of PrP^Sc^ ranged from rates consistent with both slow and fast axonal transport. Interestingly, transport of PrP^Sc^ was not dependent upon prion conversion as it was observed using prion strain and host combinations that do not support prion formation. The rate of PrP^Sc^ transport of prion strains and synthetic prions suggest that prions can use both slow and fast modes of axonal transport. Overall, converging lines of evidence suggest that prions are transported at a range of velocities consistent with both slow and fast axonal transport that is independent prion formation.

## Author summary

Prion diseases are fatal neurodegenerative diseases caused by PrP^Sc^, the self-templating conformation of the normal host-encoded prion protein, PrP^C^. Transport of PrP^Sc^ in the nervous system follows a highly predictable spatial pattern consistent with spread along defined neuroanatomical pathways. The velocity of PrP^Sc^ transport and the requirement for prion conversion during transport are poorly understood. To address this problem, we developed a method to determine the velocity of fluorescently tagged PrP^Sc^ particles in the sciatic nerve of animals. We measured the velocity of PrP^Sc^ particles that ranged from rates consistent with both slow and fast axonal transport. Additionally, we found transport of PrP^Sc^ was not dependent upon prion conversion as it was observed under conditions that do not support prion formation. Overall, these results suggest that prions are transported at a range of velocities consistent with both slow and fast axonal transport that is independent prion formation.

## Introduction

Prion diseases are invariably fatal, transmissible, neurological disorders that affect a wide variety of mammalian species. Prion diseases are unique in biology in that they can have three etiologies, sporadic, familial and infectious. Prions are comprised of PrP^Sc^, the self-templating conformation of the host encoded prion protein, PrP^C^ [1–5]. Prion formation occurs when PrP^C^ binds to PrP^Sc^ and, through an unknown process, PrP^Sc^ directs a global rearrangement of the helical structure of PrP^C^ to a parallel in register intermolecular beta sheet structure of PrP^Sc^ [6–8]. Fragmentation of the growing PrP^Sc^ fibril results in generation of new free ends that support prion formation [9,10]. Repeated cycles of PrP^Sc^ formation and fibril fragmentation can result in exponential amplification of PrP^Sc^ and prion infectivity that is thought to be recapitulated *in vitro* using protein misfolding cyclic amplification (PMCA) [11–13].

Prion pathogenesis is characterized by prion spread in the nervous system [14–17]. Following intraperitoneal and oral routes of infection, prions can establish infection in Peyer’s patches, gut-associated lymphoid tissues, spleen and lymph nodes [18–21]. From these tissues, through an unknown process, prions gain access to sympathetic and parasympathetic nerves where they are transported to the central nervous system [22–26]. Direct inoculation of peripheral nerves (e.g., optic, sciatic nerve) results in spread of PrP^Sc^ along synaptically connected neuroanatomical pathways that include peripheral ganglia, peripheral nerves (somatic and autonomic), spinal cord tracts, and brainstem nuclei [27–33]. Overall, prions are transported along synaptically connected structures where they can cause neurotoxicity, resulting in neuronal death.

The rate of prion transport within neural pathways is poorly understood. Initial studies tracked prion spread using the presence of vacuolation pathology or infectivity of target tissues, leveraging the predictable pattern of neuroanatomical spread of PrP^Sc^. The rate of prion infectivity is consistent with slow axonal transport at a rate of approximately 0.8-4.0 mm/day (0.01-0.05 µm/s) that was calculated after inoculations via the ocular, intraperitoneal, sciatic, or oral routes of infection [29,34–36]. Subsequent studies utilizing immunohistochemistry or immunoblot detection of PrP^Sc^ similarly observed that the rate of PrP^Sc^ spread was consistent with slow axonal transport [37]. Additionally, the rate of PrP^Sc^ spread was measured between three well-characterized hamster-adapted prion strains, the hyper (HY) strain of hamster-adapted transmissible mink encephalopathy (TME), the drowsy (DY) strain of hamster-adapted TME and the 139H strain of hamster-adapted scrapie. These three hamster-adapted prion strains differ in incubation period, clinical signs and distribution of PrP^Sc^ and/or pathology in the host [38–41]. Interestingly, strain-specific rates of PrP^Sc^ spread were observed between HY TME, DY TME, and 139H suggesting strain-specific differences in axonal transport [42–44]. The methods used in the aforementioned studies, however, cannot distinguish between the inoculated PrP^Sc^ and newly formed PrP^Sc^. It is possible, therefore, that the previously reported PrP^Sc^ velocities are instead a combination of both PrP^Sc^ transport and conversion where PrP^Sc^ is only identified once it has accumulated above the detection threshold [17].

Overall, bona fide axonal transport of PrP^Sc^ particles has not been observed or measured directly. In this study, we investigated the rate of PrP^Sc^ axonal transport, the effect of prion strain on PrP^Sc^ velocity, and if transport requires prion formation..

## Materials and methods

### Ethics statement

All procedures involving animals comply with the *Guide for the Care and Use of Laboratory Animals* and were approved by the Creighton University Institutional Animal Care and Use Committee.

### Prion inocula

Brains from terminally-ill mice or hamsters infected with either the RML, 263K, HY TME, DY TME or 139H strains of prions were collected and homogenized to 10% w/v in Dulbecco’s phosphate buffered saline (DPBS).

### Sciatic nerve inoculation, tissue collection and processing

Inoculation of the sciatic nerve (ScN) was performed as previously described [44]. Briefly, mice were anesthetized and maintained in the plane of anesthesia with inhaled isoflurane. The right ScN was exposed using sterilized surgical instruments and separated from the surrounding fascia and musculature. Closed spring scissors were used to bring the nerve into the superficial field out of the surrounding structures taking care not to damage the nerve, and a 30-gauge Hamilton syringe (Hamilton Company, Reno, NV) was utilized to inject 1 µl of the prion sample into the sciatic nerve after the needle had penetrated the epineurium. The nerve was returned to its location within the musculature, and the surgical incision was closed with surgical staples. Mice were then given 30 µl of butorphanol (2 mg/kg; Patterson Veterinary, Loveland, CO) and allowed to recover in their home cage. At 24 h post infection (p.i.), mice were anesthetized with isoflurane and transcardially perfused with DPBS. Both the inoculated (ipsilateral) and the uninoculated (contralateral) ScN were removed. The lumbar (L3-L4 vertebral segments) spinal cord (SC) was also removed from the vertebral column. To limit cross contamination, tissues were harvested from the least infectious to the most infectious (in order: contralateral ScN, lumbar SC, ipsilateral ScN). Tissues were frozen at -80^o^C.

### Protein misfolding cyclic amplification

Protein misfolding cyclic amplification (PMCA) was performed as previously described [45]. Briefly, dissected tissues were homogenized in a bead mill homogenizer (OMNI International, Kennesaw, GA) in sterile DPBS to produce 10% w/v tissue homogenates. In a thin-walled PCR tube, 5 µl of tissue homogenates were seeded into 45 µl of 10% w/v uninfected (Un) hamster or Un mouse brain homogenate (BH) in PMCA conversion buffer (1% Triton X-100, 1 tablet Complete protease inhibitor, 6 mM EDTA, 150 mM NaCl, 100 µg/ml Heparin, 0.05% Digitonin, sterile DPBS) before being subjected to 2 rounds of PMCA using a Q700 sonicator with a microplate horn (Qsonica, Newtown, CT). In the second serial round of PMCA, 5 µl of the round 1 PMCA reaction was added to 45 µl of Un hamster BH in PMCA conversion buffer. Each round of PMCA had a duration of 72 h at 37^o^C with 1 second of active sonication every 10 min (rest for 9:59 min). Sonicator amplitude was set at 37–40 with a wattage of 220–280 W. Unseeded samples containing only Un hamster BH in PMCA conversion buffer served as negative controls, and HY TME BH dilutions seeded into Un hamster BH in PMCA conversion buffer served as positive controls for each PMCA experiment. All samples were seeded in triplicate.

### Western Blot analysis

Western blot analysis was performed as previously described [45]. Briefly, 10 µl of PMCA reaction was combined with 10 µl of proteinase K (PK; 0.05 mg/ml; MilliporeSigma, Burlington, MA), and the reaction was incubated at 37^o^C with constant shaking. Afterwards, 20 µl of 2x sample buffer (4% w/v SDS, 2% v/v β-mercaptoethanol, 40% v/v glycerol, 0.004% w/v Bromophenol blue, and 0.5 M Tris buffer pH 6.8) was added to the reaction mixture and incubated at 100^o^C for 10 min. The reaction mixture was placed on ice, and 10 µl of the mixture was size fractionated on 4–12% Bis-Tris NuPage polyacrylamide gels (Invitrogen, Carlsbad, CA). Gels were transferred onto polyvinylidene difluoride (PVDF) membranes (MilliporeSigma, Burlington, MA). Membranes were blocked with 5% w/v blotto (Bio-Rad Laboratories, Hercules, CA) in 0.05% v/v tween tris-buffered saline (TTBS; BioRad Laboratories, Hercules, CA) and then incubated overnight with anti-PrP antibody 3F4 or anti-PrP 8H4 monoclonal anti-PrP primary antibody (1:10000). Membranes were then washed five times in TTBS and incubated in goat anti-mouse HRP conjugated secondary antibody for 1 hour (1:4000). Membranes were then washed five times in TTBS, exposed with ECL reagent, and imaged via chemiluminescence in the Li-Cor Odyssey XF imager (Li-Cor Biosciences, Lincoln, NE).

### Production of infectious synthetic mouse PrP^Sc^

Spontaneous generation of recombinant *bona fide* prions was performed as described previously [46,47]. Briefly, 100 mg of acid-washed 1 mm glass beads (Sigma-Aldrich) were placed in clean, labelled 2-ml tubes with screw caps, containing 500–800 µl of fresh substrate. Protein misfolding shaking amplification (PMSA) was performed for 24 h, 39°C and with continuous shaking at 700 rpm using programmable thermoblocks (Digital Shaking Drybath, Thermo Scientific). Up to four 24 h serial PMSA rounds were performed, diluting 1:10 the PMSA product from the previous round into a new tube with freshly thawed rec mouse PrP^C^ substrate and their corresponding glass beads. To produce the Mo recPrP^Sc^ batch employed in this study, a two-step propagation was performed by PMSA. First, four 2-ml screw cap tubes with 1 ml of fresh PMSA substrate each, were complemented with three clean zirconia-silicate beads of 2.3 mm (BioSpec Products Inc., Bartlesville, OK) and 3 prion-coated beads, that were subjected to PMSA for 5 h. Then, the product of this first round was used at 1:5 dilution to seed 5-ml screw cap tubes each containing 4 ml of fresh PMSA substrate and 15 clean zirconia-silicate beads of 2.3 mm. These were then submitted to a 19-h PMSA reaction. The product of this reaction was analysed by electrophoresis and total protein staining to confirm efficient propagation and conservation of the original electrophoretic mobility pattern.

### PrP^Sc^ enrichment

Enrichment of PrP^Sc^ from BH was performed as previously described [48]. Briefly, 2 μl of PK (2.5 mg/mL) was added to 200 μl aliquots of 10% w/v BH from animals terminally infected with various prion strains and incubated with shaking at 37^o^C for 30 min. Afterwards, 4.1 μl of 0.5M EDTA (pH 8.0), 206 μl of 4% w/v sarkosyl in DPBS, and 0.83 μl of benzonase (MilliporeSigma, Burlington, MA) were added and were mixed by inverting the tube 10 times and incubated with shaking at 37^o^C for 10 min. Subsequently, 33.5 μl of 4% w/v sodium phosphotungstate in ultrapure H_2_O (NaPTA; pH 7.4) was added, mixed by inverting the tube 10 times, and incubated with shaking at 37^o^C for 30 min. Then, 705.3 μl iodixanol and 57.2 μl of NaPTA was added. Samples were mixed by inverting the tube 10 times, and centrifuged at 16,100g for 90 min. After centrifugation, 500 μl of the clarified supernatant was added to a durapore-PVDF 0.45 μm filtration column (Merck Millipore. Burlington, Massachusetts) and centrifuged at 12,000g for 30 s (each starting tube generated two 500 μl aliquots). The filtered sample (~480 μl) was combined with 480 μl of 2% w/v sarkosyl/0.3% w/v NaPTA in ultrapure H_2_O (pH 7.4), samples were mixed by inverting the tube 10 times and incubated with shaking at 37^o^C for 10 min. Samples were then centrifuged at 16,100g for 90 min. After centrifugation, the supernatant was removed, and the pellet was resuspended in 10 μl of wash buffer (17.5% iodixanol and 0.1% sarkosyl in DPBS). Samples were sonicated in a cup horn sonicator at 50% power for 10 s twice with resting on ice for 15 s in between. Split samples were pooled into a single 20 µl aliquots, and 180 μl of wash buffer and 16.2 μl of 4% w/v NaPTA was added. Samples were mixed by inverting the tube 10 times, and centrifuged at 16,100g for 30 min. The supernatant was removed, and samples were resuspended in 200 μl of wash buffer and 16.2 μl of 4% w/v NaPTA. Samples were again mixed by inverting the tube 10 times, and centrifuged at 16,100g for 30 min. The supernatant was removed, and samples were resuspended in 20 μl of 0.1% sarkosyl in DPBS. To concentrate the PrP^Sc^, 12 purified 20 µL PrP^Sc^ aliquots in 0.1% sarkosyl from the previous step were combined and centrifuged for 30 min at 16,100g. The supernatant was removed, and samples were resuspended in 20 μl of 0.1% sarkosyl. Successful isolation of PrP^Sc^ was confirmed by WB, and the purity of PrP^Sc^ was determined by Sypro Ruby (Thermo Fisher Scientific, Waltham, MA) staining of size fractionated PrP^Sc^ following the manufacturer’s protocol. Gels were imaged with the Cytivia typhoon scanner (Marlborough, MA) using the Cy3 laser and filter set (excitation: 532 nm, emission: 560–580 nm, PMT: 500–600 V).

### Fluorophore conjugation

Enriched brain derived or synthetic PrP^Sc^ was labeled with Alexa Fluor 647 dye (AF^647^; Thermo Fisher Scientific, Waltham, MA) or pHrodo Deep Red tetrafluorophenyl (TFP) ester (pHrodo; Thermo Fischer Scientific, Waltham, MA) according to the outlined protocol. Briefly, 20 μl of enriched, concentrated PrP^Sc^ was combined with 12 μl of AF^647^ dissolved in DMSO, incubated at room temperature for 1 hour with shaking, and then incubated at 4^o^C for 48–72 h. Samples were shielded from light at all steps. After incubation, samples were centrifuged for 30 min at 16,100g and the supernatant containing unconjugated AF^647^ was removed. The pellet was resuspended in 100 μl of 10 mM glycine to quench any remaining unconjugated AF^647^ and centrifuged for 30 min at 16,100g. This was repeated two more times, and the final pellet was resuspended in 20 μl of 10% sarkosyl in DPBS. Successful conjugation of PrP^Sc^ was confirmed by fractionating the samples on 4–12% Bis-Tris NuPage polyacrylamide gels and imaging with the Cytivia Typhoon scanner using the Cy5 laser and filter set (excitation: 635 nm, emission: 655–685 nm, PMT: 500 V).

### Live sciatic nerve explant imaging

Sciatic nerve explant imaging was adapted from protocols described previously [49,50]. Anesthetized male or female FvB or PrP-/- on FvB background mice [51] were bilaterally inoculated in the ScN with either 1–2 μl of, DPBS, unconjugated AF^647^ dye, purified uninfected brain homogenate conjugated to AF^647^ (Un-AF^647^), or purified PrP^Sc^ conjugated to AF^647^ (PrP^Sc^-AF^647^) or to pHrodo (PrP^Sc^-pHrodo). The ScN was excised and placed into an FCS3 live cell chamber (Bioptechs, Butler, PA) heated and maintained at 37^o^C, and perfused continuously with oxygenated glucose solution (98 mM NaCl, 1 mM KCl, 2 mM KH_2_PO_4_, 1 mM MgSO_4_, 1.5 mM CaCl_2_, 5.6 mM D-glucose, 24 mM NaHCO_3_, 95% O_2_/5% CO_2_). The ScN samples were imaged starting approximately 20 minutes after inoculation on a Leica TCS SP8 multiphoton confocal microscope (Leica Microsystems, Wetzlar, Germany) using the Mai-Tai Deep-See ultrafast laser (Spectra-Physics, Milpitas, CA) as an excitation source (excitation: 840 nm). The emission bandpass was set to detect from 660-720 nm to encompass the peak emission of AF^647^. Using an HC PL APO 63X/1.4 oil immersion objective, Second Harmonic Generation (SHG) from collagen at 420 nm was used to localize the nerve and bring the axons into focus, and then confocal/multiphoton excitation was used to focus on groups of axons containing fluorescent particles. Axons were identified as the structures containing fluorescent particles that ran parallel to the body of the nerve and spanned the imaging field. Fluorescence specific to the AF^647^ labeled PrP^Sc^ was confirmed through lambda spectral scans (excitation: 840 nm; emission: 600–750 nm, consecutive 10 nm intervals). Time series images were acquired for 1–3 min at 63X with 3–5 s intervals between consecutive frames.

Time series images were analyzed using the Fiji/ImageJ plugin, Trackmate [52]. The smooth function was used to reduce noise, and regions of interest (ROI) were drawn around individual axons in the first frame of the time series. Particles were detected utilizing the DOG algorithm which is suitable for particles < 5 μm in diameter. Most particles observed within axons were fine and punctate with diameters < 5 µm. Particle diameter was set to 1 μm to ensure all possible particles in the axon were detected, and particles with a quality score at or above 5 were selected for track analysis. Most particles with a quality score > 5 were within the main body of the axon and displayed strong fluorescent signal that could be easily tracked. The nearest neighbor tracking algorithm was used and the distance between frames was set to the maximal possible distance a particle could travel in the given frame interval. Track velocity data was saved in Microsoft Excel. The mean velocity of each particle > 0.1 μm/sec was selected and analyzed statistically.

### Live animal sciatic nerve imaging

Male FvB/N or PrP^-/-^ mice on a FvB background were anesthetized with inhaled isoflurane and unilaterally inoculated in the sciatic nerve as described above [51]. A triangular piece of parafilm was slid between the lifted nerve and the muscular fascia to physically separate the sciatic nerve from the body plane of the animal. Mice were intraperitoneally anesthetized with a ketamine/xylazine cocktail (87.5/12.5 mg/kg) using an insulin syringe. To stabilize the anesthetized animal, the animal was placed onto the adjustable platform of a purpose-built live animal imaging system with the exposed nerve in contact with the coverslip. The platform was maintained at 37^o^C by a temperature control unit (TC 324B, Warner Instruments, Holliston, MA). The ScN of the live mouse was imaged beginning approximately 30 minutes after inoculation to identify fluorescent particles using the Leica SP8 multiphoton microscope, and the images were analyzed as described above.

### Statistical analysis

A multifactorial ANOVA was performed comparing PrP^Sc^ particle velocities in sciatic nerve explants between strains, mouse PrP genotype, and mouse sex (4 strains x 2 genotype x 2 sex). A multifactorial ANOVA was performed comparing PrP^Sc^ particle velocities for live animal imaging between strains and mouse PrP genotype (5 strains x 2 genotype). To reduce the possibility of statistical significance solely due to large n in each group, all recorded particle velocities > 0.1 µm/s were averaged within each axon to get a single average particle velocity for each individual axon. A White-Huber correction was done to account for heteroscedasticity, and significant main effects were probed with Fisher’s LSD post hoc tests. All omnibus and post hoc tests were considered significant if p < 0.05.

## Results

### Spatiotemporal detection of inoculum PrP^Sc^ in anatomically connected locations consistent with spread by fast axonal transport

**Fig 1:**
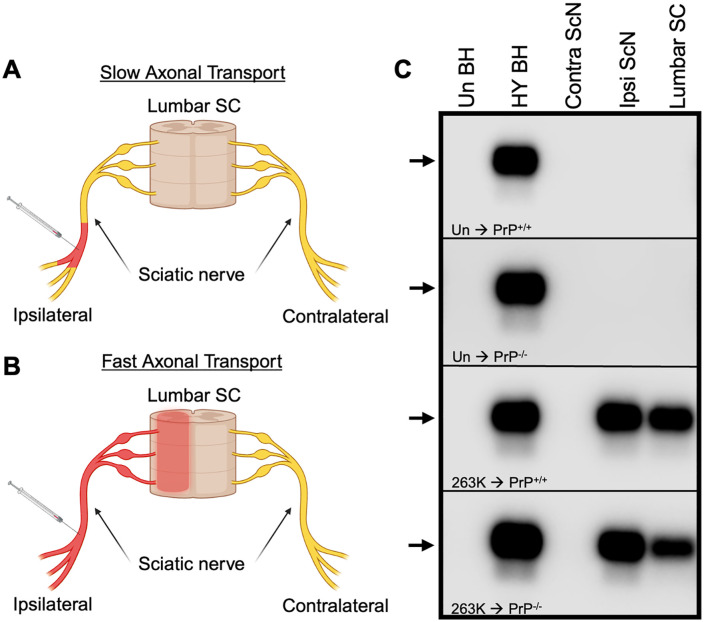
Temporal spread of inoculum PrP^Sc^ from the site of inoculation in the sciatic nerve to the lumbar spinal cord. Predicted spread of PrP^Sc^ following sciatic nerve inoculation via either A) slow or B) fast axonal transport rates at 24 h post sciatic nerve inoculation. C) Western blot analysis of proteinase K digested PMCA reactions of sciatic nerve PrP^+/+^ (1^st^ and 3^rd^ panel from the top) or PrP^-/-^ mice (2^nd^ and 4^th^ panel from the top) inoculated with either uninfected (UN, top two panels) or 263K-infected hamster brain homogenate (bottom two panels) at 24 h post inoculation. Arrows indicate the migration of the 29 kDa molecular weight marker. Created with BioRender. www.biorender.com.

The distance from the site of inoculation in the sciatic nerve to the lumbar spinal cord was determined as previously described [44]. Briefly, the spinal cord was exposed by removal of all vertebral laminae and the site of sciatic nerve inoculated was dissected proximally to the location at which the spinal nerves entered the intervertebral foramen and followed proximally in the vertebral canal to the location at which they entered the spinal cord. The distance between the site of inoculation in the sciatic nerve and where the sciatic nerve entered the spinal cord is 25mm. Following sciatic inoculation of prions, if PrP^Sc^ utilized slow axonal transport, PrP^Sc^ would be detected in the ipsilateral sciatic nerve and not in the lumbar spinal cord at 24 h postinfection (Fig 1, Panel A). Alternatively, if PrP^Sc^ utilizes fast axonal transport, inoculum PrP^Sc^ would be detected in the lumbar spinal cord in addition to the ipsilateral sciatic nerve (Fig 1, Panel B).

The PMCA protocol used was able to detect as little as 5 x 10^-12^ μg eq of starting HY infected brain homogenate seeded into uninfected hamster brain homogenate substrate after two rounds of PMCA (S1 Fig, Panel A). In contrast, similar amounts of HY TME did not seed uninfected mouse brain homogenate after two serial rounds of PMCA (S1 Fig, Panel B). Our unseeded negative controls, containing only uninfected hamster or mouse PMCA substrate, did not amplify PrP^Sc^ after two serial rounds of PMCA (S1 Fig).

As negative controls, PrP^+/+^ or PrP^-/-^ mice were inoculated in the sciatic nerve with uninfected hamster brain homogenate (Fig 1, Panels A, B). Following two serial rounds of PMCA, PrP^Sc^ was not detected in the ipsilateral sciatic nerve, the lumbar spinal cord or the contralateral sciatic nerve, from either the PrP^+/+^ or PrP^-/-^ mice (Fig 1, Panel C). Sciatic nerve inoculation of PrP^+/+^ mice with 263K prions resulted in PMCA detection of PrP^Sc^ in the ipsilateral sciatic nerve and lumbar spinal cord, but not in the contralateral sciatic nerve (Fig 1, panel C). Although there is a large species barrier between 263K hamster prions and mice as determined by PMCA (S1 Fig, Panel B) and bioassay (S5 Fig), and the 3F4 anti-PrP antibody recognizes hamster, but not mouse PrP, we could not formally exclude the possibility that detected PrP^Sc^ is newly formed PrPSc rather than inoculum. To exclude this possibility, 263K prions were inoculated into the sciatic nerve of PrP^-/-^ mice, which do not support prion formation, that resulted in PMCA detection of PrP^Sc^ in the ipsilateral sciatic nerve and lumbar spinal cord, but not in the contralateral sciatic nerve at 24 h postinfection (Fig 1, panel C). For each group, a minimum of three mice were examined with similar results. Overall, the rate of PrP^Sc^ transport was at least 25 mm/day.

### Purification of PrP^Sc^ and conjugation to AF^647^ retains prion infectivity

PrP^Sc^ from multiple prion strains were purified from hamster and mouse brain homogenates and the purified PrP^Sc^ was size fractionated on SDS-PAGE and stained with Sypro Ruby (S2 Fig). The bands <25 kDa correspond to PrP^Sc^ glyco-isoforms including the strong di-glycosylated form as well as the mono-glycosylated and un-glycosylated forms (S2 Fig). Next, the purified PrP^Sc^ conjugated to AF647 dye were size fractionated on SDS-PAGE and imaged on a Typhoon imager, revealing detection of fluorescent bands corresponding to the di-, mono-, and unglycosylated forms of PrP^Sc^ conjugated to AF^647^ (PrP^Sc^-AF^647^) (S3 Fig). To confirm biological activity of PrP^Sc^-AF^647^, 10-fold dilutions of HY PrP^Sc^-AF^647^ were seeded into PMCA reactions (S4 Fig). HY PrP^Sc^-AF^647^ were able to template efficient conversion after 2 rounds of PMCA (S4 Fig). Overall, the purified PrP^Sc^ retains biological activity following conjugation to AF^647^. To investigate if this material was infectious, uninfected mouse BH, purified uninfected mouse BH, RML-infected BH, and purified RML were unilaterally inoculated into mouse sciatic nerve. All (n = 5) RML BH and purified RML preparation inoculated mice succumbed to prion disease in 146 ± 4 and 145 ± 4 days postinfection respectively (S5 Fig, Panel A). The incubation periods were not statistically different (p > 0.05). All (n = 5) mice inoculated with either the uninfected mouse BH or purified uninfected mouse BH did not develop clinical signs of prion disease by 600 days postinfection (S5 Fig, panel A). Western blot analysis of PK digested brain homogenate from each of these groups were consistent with the clinical diagnosis of disease (S5 Fig, panel B).

### Determination of PrP^Sc^-AF^647^ axonal transport velocity in PrP^+/+^ mouse sciatic nerve explant cultures

After sciatic nerve inoculation of DPBS, unconjugated AF^647^, and Un-AF^647^, individual axons could not be demarcated with fluorescent particles, and the distribution of fluorescence was random (S6 Fig). Lambda spectral scans of these random particles did not have a distinct peak in fluorescence intensity and were easily distinguishable from the lambda spectral scan pattern of AF^647^ (S6 Fig) and indicated that the low molecular weight fluorescent material that was detected in the Sypro Ruby gel of the uninfected preparations (S3 Fig) did not contribute to measurable signal in the sciatic nerve explant cultures.

**Fig 2:**
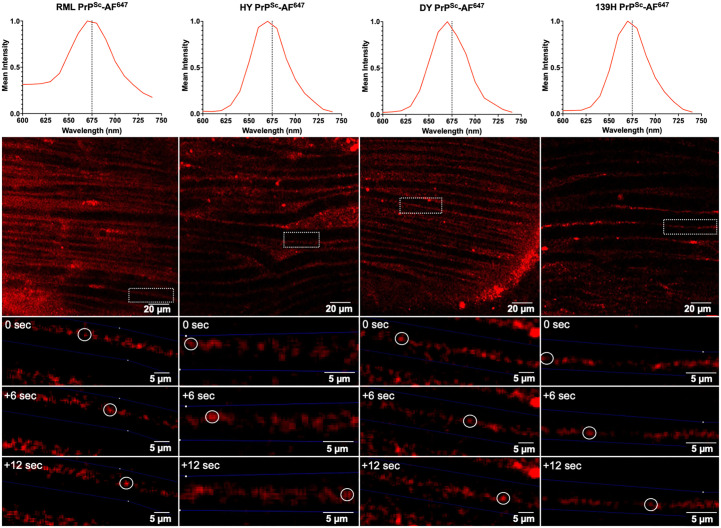
Time series images of PrP^Sc^-AF^647^ in sciatic nerve explant cultures. Murine sciatic nerve explants were inoculated with either Rocky Mountain Laboratory (RML) prions, hyper (HY), drowsy (DY), or 139H PrP^Sc^ conjugated to AF^647^. A particle is followed over 12 s with 3 selected frames showing its movement along the axon (3 s/frame). Lambda scans (top row) were consistent with AF^647^ bound particles. Still images of the nerve containing multiple axon fibers (second row from the top) with AF^647^ bound particles, and example motile particles within an individual axon (bottom three rows) were used to measure the velocity of the AF^647^ conjugated PrP^Sc^. Scale bars represent either 20 (top row) or 5 μm (bottom three rows).

**Fig 3:**
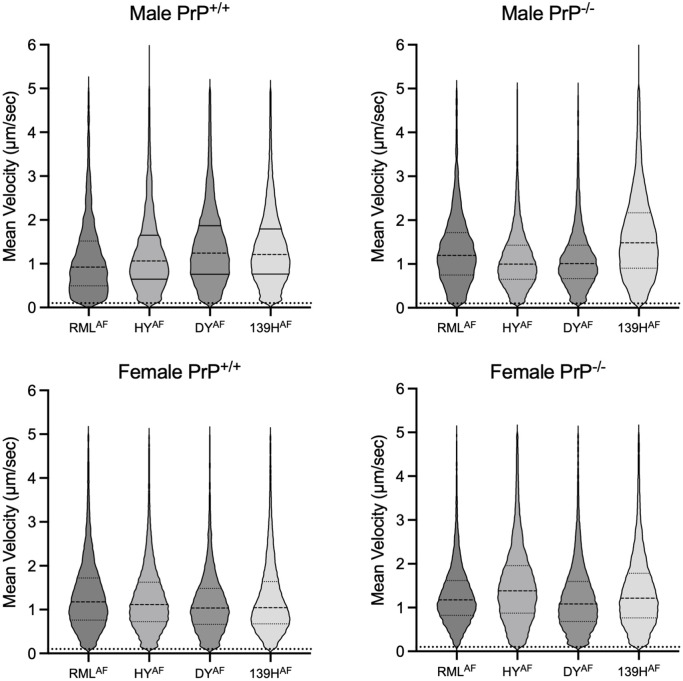
Velocities of PrP^Sc^-AF^647^ particles in sciatic nerve explant cultures. Violin plot representation of PrP^Sc^ particle velocities from either male or female PrP^+/+^ or PrP^-/-^ mice inoculated in the sciatic nerve with either, Rocky Mountain Laboratory (RML) prions, hyper (HY), drowsy (DY), or 139H PrP^Sc^-AF^647^.

To investigate PrP^Sc^ axonal transport, purified RML, HY, DY, or 139H PrP^Sc^ strains conjugated to AF^647^ were injected into the sciatic nerve and the explant was imaged using two photon confocal microscopy. Fluorescence, with all prion strains examined, in both PrP^+/+^ and PrP^-/-^, and in both male and female animals, localized to multiple sciatic nerve axons within the imaging field and throughout the nerve body (Fig 2). Lambda scans confirmed the presence of PrP^Sc^-AF^647^ with an intensity peak at ~675 nm (Fig 2). Particles from each strain had a heterogenous size distribution that contained fine particles and larger, brighter aggregates (Fig 2). Within each axon, multiple particles were localized by Trackmate and tracked over multiple frames to calculate mean velocity. To investigate PrP^Sc^ transport, we assembled a distribution profile of PrP^Sc^ particle velocities between the prion strains, host genotype and animal sex (Fig 3) and represented the average velocities of these particles, plus the percentage of the total particles that had a measured mean velocity of > 2 μm/sec (Table 1) and compared statistically (S1 Table). For each group, a minimum of three mice were examined and velocities of a minimum of 5000 particles were measured.

**Table 1:** Average velocities of PrP^Sc^ particles in mouse sciatic nerve explant cultures.

		Male		Female	
PrP Genotype	Prion Strain	Mean velocity (µm/s)^a^	Percentage >2 µm/s^b^	Total velocity (µm/s)	Percentage >2 µm/s
PrP ^+ /+^	RML	1.11±0.0488	13.9%	1.35±0.0281	17.0%
	HY TME	1.30±0.0342	17.0%	1.27±0.0152	14.2%
	DY TME	1.38±0.0270	21.5%	1.18±0.0208	11.0%
	139H	1.39±0.0215	19.3%	1.46±0.0366	16.3%
PrP^-/-^	RML	1.34±0.0207	16.6%	1.31±0.0223	13.2%
	HY TME	1.13±0.0180	10.0%	1.55±0.0185	23.6%
	DY TME	1.15±0.0232	10.4%	1.25±0.0213	13.9%
	139H	1.73±0.0248	30.1%	1.41±0.0292	19.1%

a Mean velocity of total PrP^Sc^-AF^647^ measured ± standard error of the mean

b Percentage of all PrP^Sc^-AF^647^ measured (>0.1 µm/sec) with a velocity of >2 µm/sec

### Determination of PrP^Sc^-AF^647^ axonal transport velocity in the intact sciatic nerve of live mice

**Fig 4:**
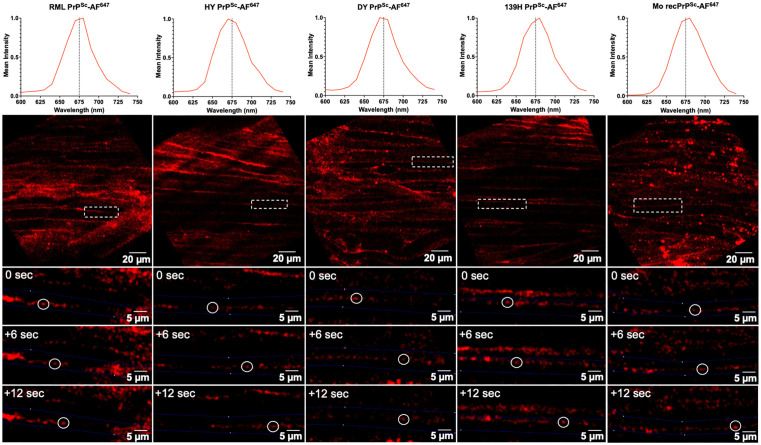
Time series images of PrP^Sc^-AF^647^ in the sciatic nerve of live animals. Murine sciatic nerve explants were inoculated with either Rocky Mountain Laboratory (RML) prions, hyper (HY), drowsy (DY), 139H or synthetic mouse recombinant (Mo rec) PrP^Sc^ conjugated to AF^647^. A particle is followed over 12 s with 3 selected frames showing its movement along the axon (3 s/frame). Lambda scans (top row) were consistent with AF^647^ bound particles. Still images of the nerve containing multiple axon fibers (second row from the top) with AF^647^ bound particles, and example motile particles within an individual axon (bottom three rows) were used to measure the velocity of the AF^647^ conjugated PrP^Sc^. Scale bars represent either 20 (top row) or 5 μm (bottom three rows).

**Fig 5:**
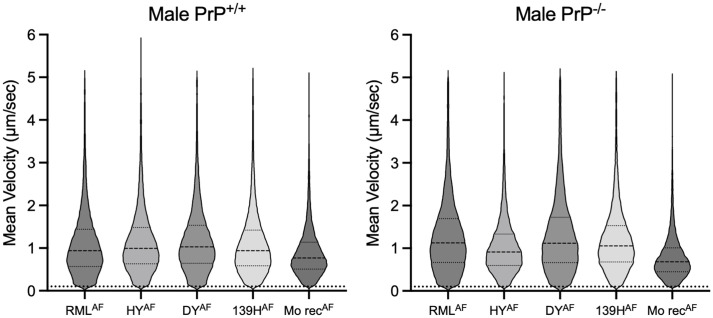
Velocities of PrP^Sc^-AF^647^ particles in the sciatic nerve of live animals. Violin plot representation of PrP^Sc^ particle velocities from male PrP^+/+^ or PrP^-/-^ mice inoculated in the sciatic nerve with either, Rocky Mountain Laboratory (RML) prions, hyper (HY), drowsy (DY), 139H or synthetic mouse recombinant (Mo rec) PrP^Sc^-AF^647^.

To measure PrP^Sc^ axonal velocity in live animals, the sciatic nerve was inoculated with PrP^Sc^-AF^647^ from RML, HY, DY, 139H or Mo recPrP^Sc^. Similar to what was observed in the explant culture, fluorescent particles localized to axons with a heterogenous particle size distribution and lambda scans confirmed the presence of AF^647^ (Fig 4). To investigate PrP^Sc^ transport velocities, we assembled a distribution profile of PrP^Sc^ between the prion strains and host genotype (Fig 5) and represented the average velocities of these particles plus the percentage of the total particles that had a measured mean velocity of > 2 μm/sec (Table 2) and compared statistically (S2 Table). For each group, a minimum of three mice were examined and velocities of a minimum of 5000 particles were measured.

**Table 2:** Average velocities of PrP^Sc^ particles in mouse sciatic nerve from live animals.

PrP Genotype	Prion Strain	Mean velocity (µm/s)^a^	Percentage >2 µm/s^b^
PrP ^+ /+^	RML	1.14±0.0166	11.7%
	HY TME	1.24±0.0202	12.2%
	DY TME	1.22±0.0135	13.3%
	139H	1.18±0.0177	11.1%
	Mo recPrP^Sc^	0.93±0.0146	6.0%
PrP^-/-^	RML	1.32±0.0151	16.9%
	HY TME	1.07±0.0153	8.9%
	DY TME	1.31±0.0248	18.2%
	139H	1.24±0.0213	12.6%
	Mo recPrP^Sc^	0.85±0.0167	4.7%

a Mean velocity of total PrP^Sc^-AF^647^ measured ± standard error of the mean

b Percentage of all PrP^Sc^-AF^647^ measured (>0.1 µm/sec) with a velocity of >2 µm/sec

Synthetic mouse recombinant PrP^Sc^ (Mo recPrP^Sc^) is infectious in animals [46,47]. Following sciatic nerve inoculation of Mo recPrP^Sc^-AF^647^, similar to what is observed in brain derived fluorescently labeled PrP^Sc^ in live animal sciatic nerves, the fluorescent Mo recPrP^Sc^-AF^647^ particles localized to axons (Fig 4) with similar distribution profiles and velocities (Fig 5, Table 2). As the AF^647^ labeled (Mo recPrP^Sc^-AF^647^) is only generated from PrP, this excludes the possibility that contaminants in the purified PrP^Sc^ from brain, and not PrP^Sc^, is being measured in the live animal imaging studies. A minimum of three mice were examined and velocities of a minimum of 5000 particles were measured.

**Fig 6:**
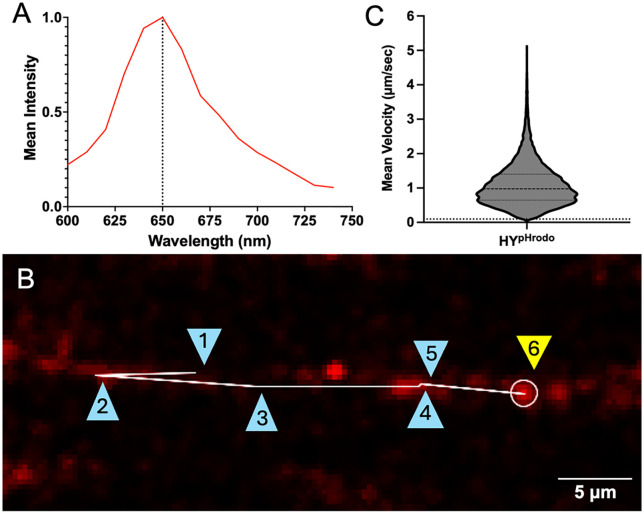
Time course image of HY TME PrP^Sc^-pHrodo labeled PrP^Sc^ particles in male PrP^+/+^ mice. A) Lambda scan of particles measured is consistent with pHrodo emission spectra. B) Image of a sciatic nerve inoculated with PrP^Sc^-pHrodo from a live animal showing the final position (number 6, yellow triangle) of an individual PrP^Sc^-pHrodo particle. The five previous positions (numbered 1 through 5, blue triangles) of the PrP^Sc^-pHrodo particle at 3 s intervals. C) Violin plot representation of PrP^Sc^ particle velocities of HY TME PrP^Sc^-pHrodo labeled PrP^Sc^ particles. Scale bar represents 5 μm.

The velocities measured for some of the particles suggested they may associate with vesicles [53]. To begin to investigate if PrP^Sc^ is utilizing vesicle movement along axons, the sciatic nerve of wild type mice was inoculated with HY TME PrP^Sc^-pHrodo, a pH-sensitive dye, only emitting fluorescent signal in acidic environments. The particles had an emission spectrum that was consistent with the emission profile of pHrodo (Fig 6, panel A). Additionally, we found evidence for PrP^Sc^ particles where the net movement of a particle was observed in one direction (Fig 6, panel B, triangle 1 to triangle 6), the particle could be observed moving in the opposite direction (Fig 6, panel B, triangle 1 to triangle 2) and then resume movement in the original direction (Fig 6, panel B, triangle 2 to triangle 3). Finally, we found the velocity profile of the particles ranged from 0.1 to 5µm/s (Fig 6, panel C) like what was measured for HY PrP^Sc^-AF^647^ (Fig 5). A minimum of three mice were examined and velocities of a minimum of 5000 particles were measured. Overall, in the sciatic nerve of live animals, both brain-derived PrP^Sc^ and synthetic murine PrP^Sc^ were transported at velocities up to 5µm/s.

## Discussion

In whole animal experiments, inoculum PrP^Sc^ was detected by PMCA in mouse lumbar spinal cord after unilateral sciatic nerve inoculation at 24 h post-inoculation (Fig 1). Because the PMCA and Western blot conditions were optimized to detect hamster and not mouse PrP^Sc^ (S1 Fig), this indicates that the hamster inoculum PrP^Sc^ was detected in the lumbar spinal cord. Additionally, PrP^Sc^ was not detected in the contralateral sciatic nerve (Fig 1). This is consistent with previous studies where, following sciatic nerve inoculation with HY TME, PrP^Sc^ was not detected in the contralateral sciatic nerve until nine weeks post infection [44]. This also suggests that the detection of inoculum PrP^Sc^ in the lumbar cord is not due to spillover. Based on these observations, we therefore hypothesize that the detection of PrP^Sc^ in the lumbar spinal cord is due to transport along sciatic nerve axons to ventral motor neurons (VMNs), consistent with our previous studies [42–44,54,55]. However, as this is an indirect measurement of prion spread between the site of inoculation and the lumbar spinal cord, we cannot exclude the possibility that inoculum PrP^Sc^ uses an alternative pathway to gain access to the lumbar spinal cord. Based on the average distance from the inoculation location in the sciatic nerve to the lumbar spinal cord, we calculated the rate of PrP^Sc^ transport to be a minimum of 25 mm/day. This calculated rate of transport is consistent with a recent report of 263K prion transport along the sciatic nerve following footpad inoculation that suggested rates of axonal prion transport reaching 50 mm/day [56]. These rates of PrP^Sc^ transport are more than 10-fold faster than what has been reported suggesting that PrP^Sc^ may be using previously unrecognized mechanisms of axonal transport [57–60]. To further explore this observation, we directly measured the velocities of fluorescently tagged PrP^Sc^ particles in the sciatic nerve of explant cultures and in live animals.

In sciatic nerve explant cultures and *in vivo* sciatic nerves, we observed velocities of PrP^Sc^-AF^647^ that ranged from 0.1 to 5 μm/sec. We did not detect fluorescent particles in the nerves of uninfected PrP^Sc^ enriched preparations conjugated to AF^647^ (negative control, S6 Fig). Importantly, the autofluorescence excitation-emission spectra (lambda scans) of negative controls were not consistent with AF^647^ (S6 Fig). This is in stark contrast to what was observed for AF^647^ labeled PrP^Sc^ where a precise emission spectrum was observed and allowed positive identification of AF^647^ labeled PrP^Sc^ (Figs 2 and 4). In sciatic nerve explant cultures and *in vivo* intact sciatic nerves inoculated with AF^647^ labeled PrP^Sc^ we reasoned that if PrP^Sc^ exclusively used the previously reported slow axonal transport rate of spread, the measured velocities would not exceed 2 µm/sec. However, we observed that approximately 20% of the PrP^Sc^ particles measured had a mean velocity exceeding 2 µm/sec that reached up to 5 µm/sec, which is inconsistent with previous studies and suggests that a subset of PrP^Sc^ utilizes fast axonal transport (Figs 3 and 5; Tables 1 and 2). However, the relative contribution of PrP^Sc^ particles transported greater than 2 µm/sec to prion pathogenesis is unknown. To use an additional method of particle detection and investigate if PrP^Sc^ is hijacking vesicle movement along axons, we used PrP^Sc^ labeled with pHrodo Deep Red TFP Ester, a pH-sensitive dye, only emitting fluorescent signal in acidic environments. This pH-sensitivity has been utilized in cultured neurons to provide mechanistic details of intracellular trafficking of pathological proteins [61,62]. We identified PrP^Sc^ particles traveling at a velocity ranging from 0.1 to 5 µm/s, with the observed particles undergoing bidirectional transport with variable run lengths between each directional change (Fig 6, panel B). These transport dynamics are consistent with vesicle-associated kinesin and dynein motors driving transport in a coordinated effort [63], however, more work is required to determine if PrP^Sc^ associates with these vesicles. Additionally, synthetic PrP^Sc^ was found to have a similar transport velocity profile as brain derived PrP^Sc^ (Fig 5). As synthetic PrP^Sc^ lacks glycosylation, this indicates that glycosylation is not required for axonal transport consistent with previous studies showing genetic removal of N-linked PrP^C^ glycosylation sites are not required for prion disease development [64,65]. However, a lower percentage of synthetic PrPSc was observed to be transported at mean velocities of >2 μm/sec compared to brain derived PrP^Sc^ (Table 2). This biological significance of this difference is unknown. Overall, in both our *in vivo* animal studies and the *in vitro* explant culture systems, multiple converging lines of evidence suggest that a subpopulation of PrP^Sc^ may utilize fast axonal transport.

Strain-specific rates of PrP^Sc^ transport were not observed. Previous studies determined a strain-specific rate of prion transport following sciatic nerve inoculation with HY having the fastest rate at 4.14 mm/day, DY having the slowest rate at 1.10 mm/day and 139H having an intermediate rate of spread of 1.80 mm/day [42,43]. Based on this previous work, we hypothesized that HY PrP^Sc^ particles should have a velocity of nearly 4 times that of DY PrP^Sc^. What was observed, however, is that all three prion strains had nearly indistinguishable transport velocity profiles (Figs 3 and 5, Tables 1 and 2). In the systems used in this study, PrP^Sc^ transport and replication are decoupled, while in our previous studies both transport and prion replication can occur [42,43]. Based on these observations, we hypothesize that the previously observed strain-specific differences in prion spread reported in hamsters is due to differences in the rate of prion formation and not due to differences in the axonal transport velocity of PrP^Sc^ [17]. Supporting this hypothesis are PMCA studies examining the rate of prion formation where HY PrP^Sc^ formation is more efficient compared to DY and 139H [45,66,67]. Alternatively, of the range of PrP^Sc^ particles measured, or of particles that were not measured in this study (e.g., PK sensitive PrP^Sc^) it is unknown which ones contribute to the strain specific differences in pathogenesis of disease. Therefore, we cannot exclude the possibility that strain-specific differences in PrP^Sc^ transport may contribute to the pathogenesis of disease in a yet undiscovered way.

In the explant and live animal axonal transport experiments, small, but significant differences in particle velocities were observed between various prion strains, host genotype and sex (S1 and S2 Tables). However, the observed differences do not have a consistent pattern across prion strain, host genotype, or sex of the animal. It is unclear if these differences are biologically significant or are due to the large number of particles compiled and analyzed, potentially allowing small variations in mean axonal transport velocity to display significance. Overall, all strains tested are transported at similar velocities, and we hypothesize that prion strains are transported using similar mechanisms.

Transport of PrP^Sc^ was independent of PrP^C^ expression. In whole animal studies, PrP^Sc^ was detected in the lumbar spinal cord at 24 h p.i. in PrP^-/-^ mice, similar to what was observed in WT mice (Fig 1). Using both the *in vitro* sciatic nerve explant culture system and the *in vivo* sciatic nerves from PrP^-/-^ mice, we detected PrP^Sc^ particle velocities similar to what was observed in WT animals from all mouse and hamster prion strains tested (Figs 3 and 5; Tables 1 and 2). These findings are in contrast with previous work indicating that PrP^C^ expression is required for axonal prion transport [68]. In these studies, intraocular (i.o) inoculation of prions into PrP^-/-^ mice containing a PrP^C^ overexpressing brain graft failed to result in the establishment of prion infection in the grafted material [68]. The authors showed that intraocular inoculation of PrP^C^ expressing mice with the PrP^C^ expressing graft resulted in prion pathology, suggesting that the graft was synaptically connected with the retino-tectal pathway. Therefore, the failure to establish infection in the PrP^-/-^ mice was not due to a lack of anatomically connected structures but due to the lack of PrP^C^. The authors suggest that PrP^C^ is required for a “domino” mechanism of prion conversion and spread along axons and/or PrP^C^ is required for crossing of synapses. The studies presented here are inconsistent with the “domino” hypothesis and instead point to the potential requirement of PrP^C^ at the synapse for transsynaptic prion transport.

The experimental paradigm used in this study has several limitations. First, the contribution of PrP^Sc^ that is detected by PMCA in the lumbar cord 24 h after sciatic nerve inoculation to subsequent transsynaptic spread and disease development is unknown. As PMCA can detect minute levels of PrP^Sc^, it is possible the amount of PrP^Sc^ that is rapidly transported to VMNs cannot establish infection and therefore does not contribute to the pathogenesis of disease [29,69]. Second, sciatic nerve inoculation is an artificial means of introducing prions to the nervous system. While sciatic nerve inoculation can cause disease and the previously measured rate of PrP^Sc^ spread from the inoculation site in the sciatic nerve to VMNs is the same as what is measured within the CNS, it is possible that the mechanisms used differ [42,43]. Third, while the purified material is infectious following sciatic nerve inoculation and the labeling does not affect PMCA seeding activity (S4 and S5 Figs), it is possible that in natural prion disease PrP^Sc^ may associate with other proteins that affect axonal transport properties that are not accounted for in this experimental paradigm [53]. Additionally, these studies do not capture the contributions of PK sensitive forms of PrP^Sc^ or newly formed PrP^Sc^ in axonal transport.

Overall, the findings reported here provide evidence for axonal transport of PrP^Sc^ that is independent of prion strain or host PrP^C^ expression. These findings provide a clearer view of prion axonal transport and clarify an important variable in understanding strain-specific differences in the tempo of prion pathogenesis. These results suggest the potential commonalities in the axonal transport mechanisms of PrP^Sc^, consistent with previous research showing the common neuroanatomical pathways of prion spread, and strain independent uptake of prions [43,70,71]. Finally, as the similarities between prion and prion-like diseases is becoming increasingly clear, it is possible that prion-like proteins may share a similar axonal transport properties [72–76].

## Supporting information

S1 Fig Sensitivity and specificity of PMCA for detection of HY TME prions. A) Western blot analysis of proteinase K digested PMCA reactions seeded with ten-fold serial dilutions of HY TME brain homogenate into uninfected (Un) hamster brain homogenate substrate after two serial rounds of PMCA. Each panel represents a technical replicate. B) Western blot analysis of proteinase K digested PMCA reactions ten-fold serial dilutions of HY TME brain homogenate in uninfected mouse brain homogenate substrate after two serial rounds of PMCA. Each panel represents a technical replicate. The Western blots in panels A and B were probed with the monoclonal anti-PrP antibody 3F4, which recognizes hamster, but not murine PrP. Arrows indicate the migration of the 29 kDa molecular weight marker.
(TIF)

S2 Fig Enrichment of PrP^Sc^ from prion-infected brain homogenates. Sypro Ruby gel stain of proteinase K (PK) digested (+) or undigested (-) brain homogenates, or enriched preparations (prep, highlighted by green box) from either A) Rocky Mountain Laboratory (RML) prions, B) hyper (HY), C) drowsy (DY), or D) 139H infected animals. Migration of the 10, 15, 20 and 25 kDa molecular weight markers is indicated on the left of the panel. The PK band is indicated by a red arrow.
(TIF)

S3 Fig Conjugation of purified PrP^Sc^ to AF^647^. Uninfected brain and brains terminally infected with either hyper (HY), drowsy (DY), 139H or Rocky Mountain Laboratory (RML) prions purified according to the protocol from Wenborn et al., 2015 [48] and conjugated to AF^647^. Purified and fluorescently conjugated isolates were gel fractionated, and fluorescence was detected by using the Typhoon laser scanning imager.
(TIF)

S4 Fig Conjugation of AF^647^ to HY PrP^Sc^ does not reduce its biological activity. Western blot analysis of proteinase K digested brain homogenates from first and second serial rounds of PMCA reactions seeded with either uninfected brain homogenate (UN) or serial dilutions of either HY TME-infected brain homogenates or HY PrP^Sc^ conjugated to Alexa Fluor 647 (HY AF^647^).
(TIF)

S5 Fig Hamster prions fail to cause disease or establish infection following sciatic nerve inoculation of mice. A) Kaplan-Meyer survival curve of mice inoculated in the sciatic nerve with either uninfected (UN) mouse brain homogenate (green circles), enriched PrP preparations (prep) from uninfected mouse brain (blue squares), rocky mountain laboratory (RML) infected mouse brain homogenate (BH)(purple triangles), enriched PrP preparations from RML-infected mouse brain (orange upside down triangles), or brain homogenate from 263K-infected hamster brain (red diamonds). Mice inoculated with either UN mouse BH, UN mouse prep or 263K-infected hamster BH failed to develop clinical signs of prion infection by 600 days post inoculation. B) Western blot analysis of proteinase K digested brain homogenates from mice infected with the inoculums listed in panel A.
(TIF)

S6 Fig Two-photon confocal microscopy analysis of negative controls. Still images from mouse sciatic nerves inoculated with either A) DPBS, B) Unconjugated AF^647^ quenched by glycine, or C) Uninfected purified preparations conjugated to AF^647^. Lambda spectral scans (D) of fluorescence from A-C are inconsistent with the AF^647^ spectral fingerprint. Scale bar represents 20 μm.
(TIF)

S1 File Uncropped gel and blot images. (PDF)

S1 Table Comparison of average axonal velocities of PrP^Sc^ strains between PrP^+/+^ and PrP^-/-^ mouse sciatic nerve explant genotypes within mouse sex. (DOCX)

S2 Table Comparison of average axonal velocities of PrP^Sc^ strains between male PrP^+/+^ and male PrP^-/-^ in live mouse sciatic nerve axons. (DOCX)
