# Supplementary figures and images for: The axonal transport velocity of prions is independent of prion formation
Source: PLoS Pathog. 2026 Jul 24;22(7):e1014456. doi: 10.1371/journal.ppat.1014456 (PMC13423175; doi:10.1371/journal.ppat.1014456)

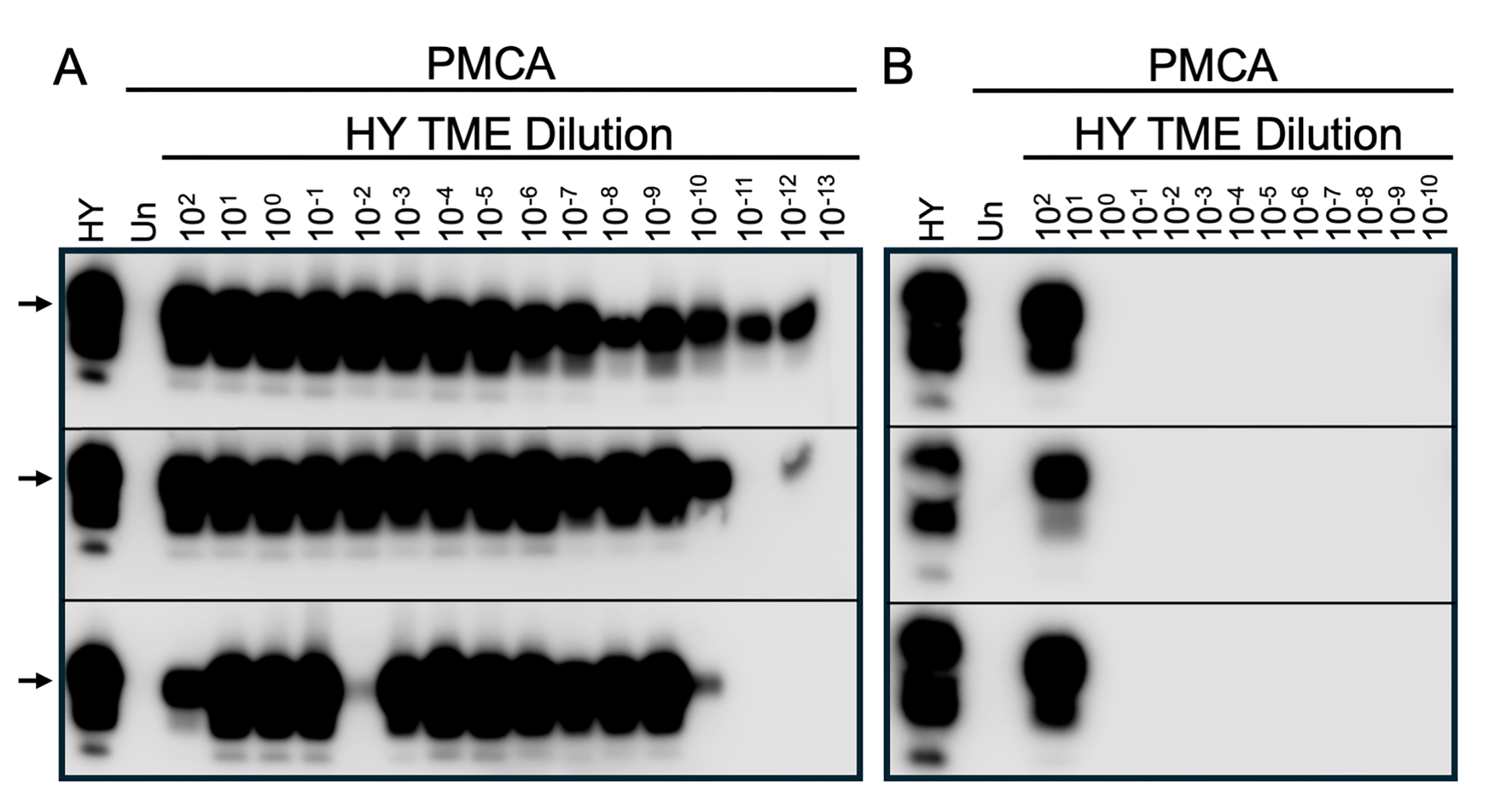

Supplement: S1 Fig — A) Western blot analysis of proteinase K digested PMCA reactions seeded with ten-fold serial dilutions of HY TME brain homogenate into uninfected (Un) hamster brain homogenate substrate after two serial rounds of PMCA. Each panel represents a technical replicate. B) Western blot analysis of proteinase K digested PMCA reactions ten-fold serial dilutions of HY TME brain homogenate in uninfected mouse brain homogenate substrate after two serial rounds of PMCA. Each panel represents a technical replicate. The Western blots in panels A and B were probed with the monoclonal anti-PrP antibody 3F4, which recognizes hamster, but not murine PrP. Arrows indicate the migration of the 29 kDa molecular weight marker. (TIF) [file ppat.1014456.s001.tif]

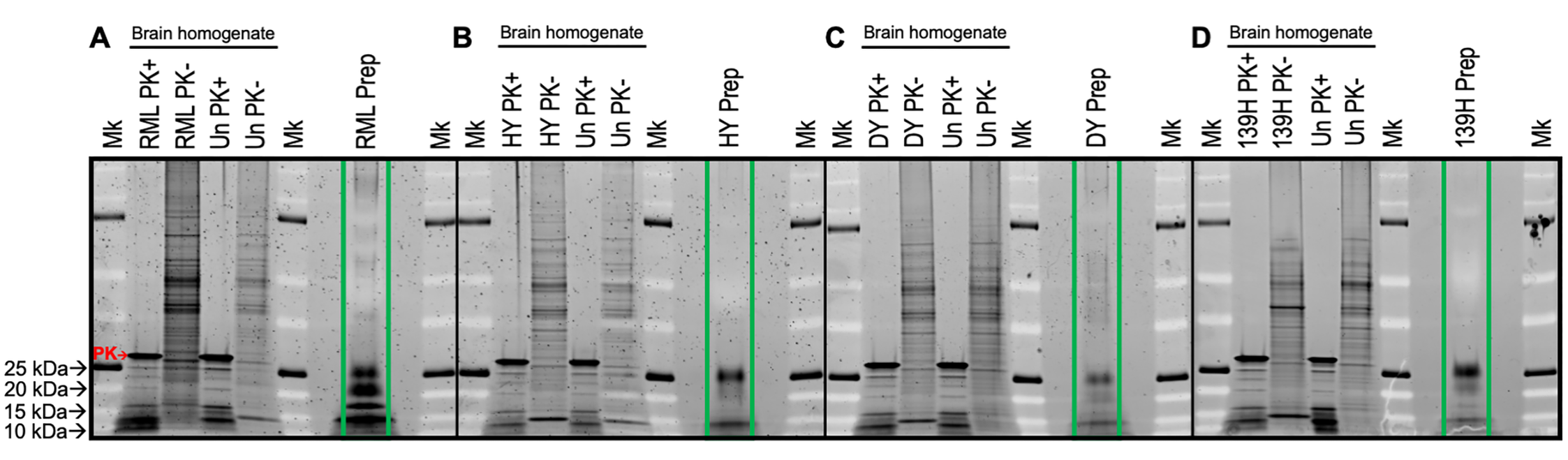

Supplement: S2 Fig — Sypro Ruby gel stain of proteinase K (PK) digested (+) or undigested (-) brain homogenates, or enriched preparations (prep, highlighted by green box) from either A) Rocky Mountain Laboratory (RML) prions, B) hyper (HY), C) drowsy (DY), or D) 139H infected animals. Migration of the 10, 15, 20 and 25 kDa molecular weight markers is indicated on the left of the panel. The PK band is indicated by a red arrow. (TIF) [file ppat.1014456.s002.tif]

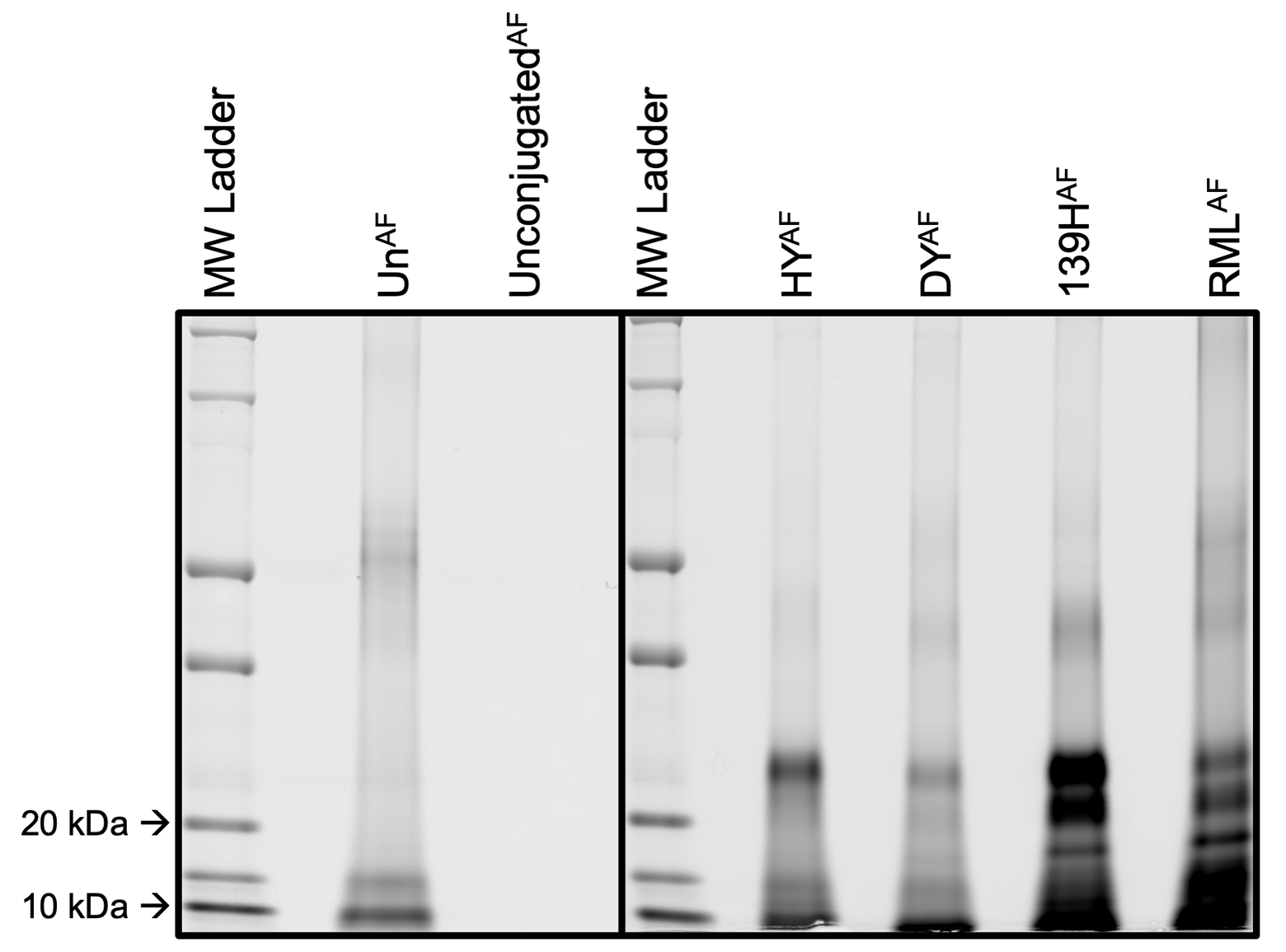

Supplement: S3 Fig — Uninfected brain and brains terminally infected with either hyper (HY), drowsy (DY), 139H or Rocky Mountain Laboratory (RML) prions purified according to the protocol from Wenborn et al., 2015 [48] and conjugated to AF647. Purified and fluorescently conjugated isolates were gel fractionated, and fluorescence was detected by using the Typhoon laser scanning imager. (TIF) [file ppat.1014456.s003.tif]

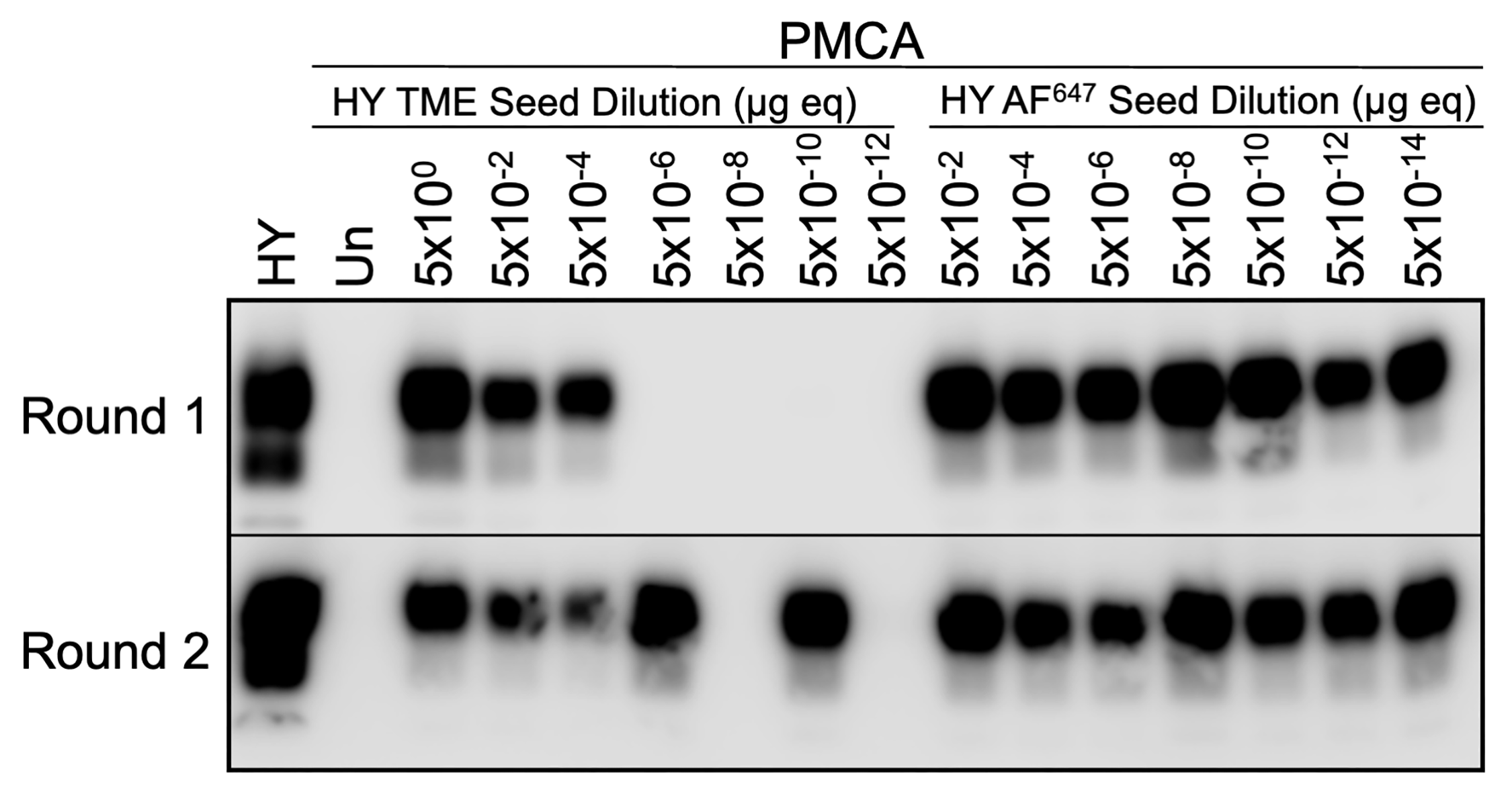

Supplement: S4 Fig — Western blot analysis of proteinase K digested brain homogenates from first and second serial rounds of PMCA reactions seeded with either uninfected brain homogenate (UN) or serial dilutions of either HY TME-infected brain homogenates or HY PrPSc conjugated to Alexa Fluor 647 (HY AF647). (TIF) [file ppat.1014456.s004.tif]

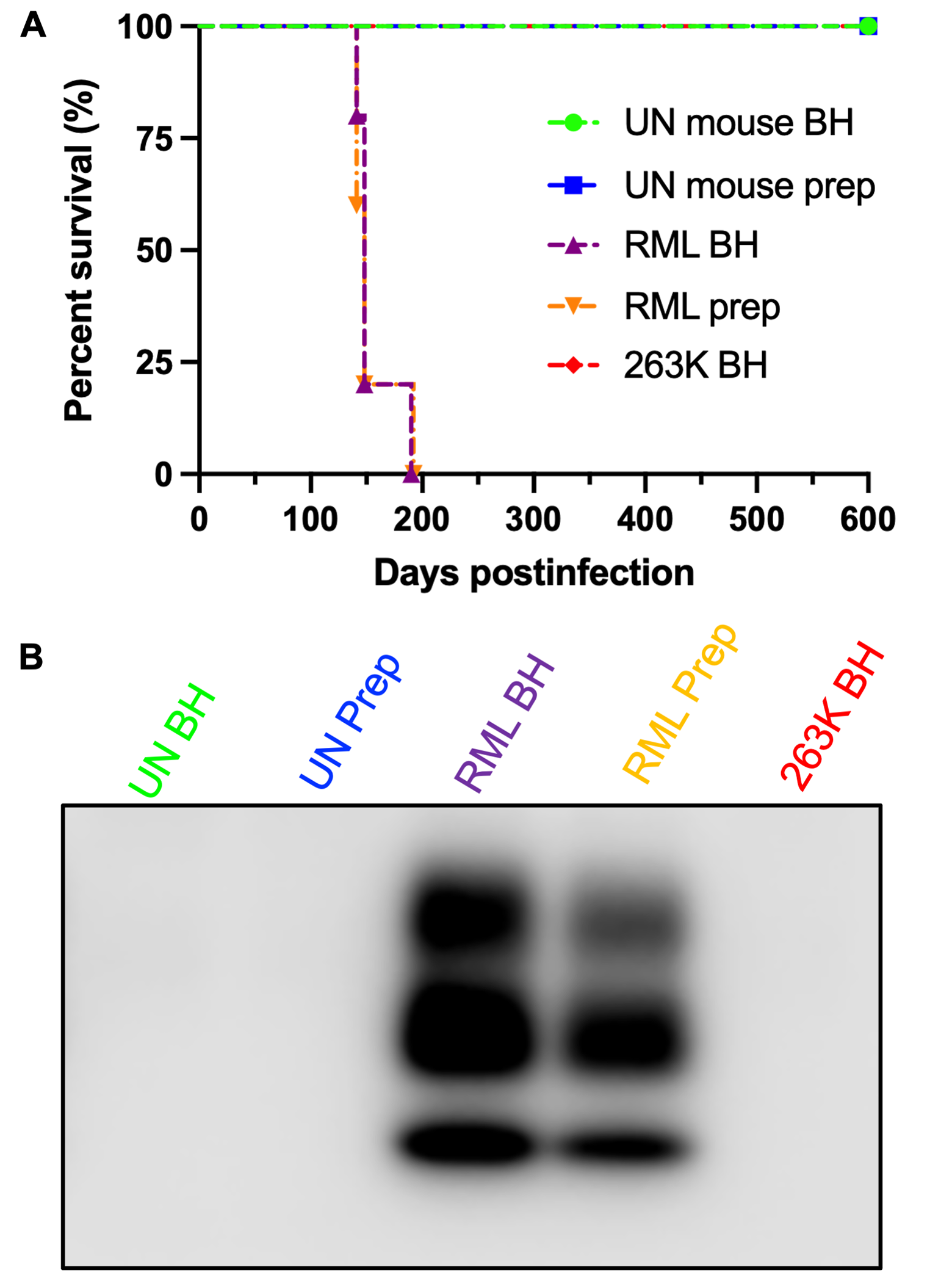

Supplement: S5 Fig — A) Kaplan-Meyer survival curve of mice inoculated in the sciatic nerve with either uninfected (UN) mouse brain homogenate (green circles), enriched PrP preparations (prep) from uninfected mouse brain (blue squares), rocky mountain laboratory (RML) infected mouse brain homogenate (BH)(purple triangles), enriched PrP preparations from RML-infected mouse brain (orange upside down triangles), or brain homogenate from 263K-infected hamster brain (red diamonds). Mice inoculated with either UN mouse BH, UN mouse prep or 263K-infected hamster BH failed to develop clinical signs of prion infection by 600 days post inoculation. B) Western blot analysis of proteinase K digested brain homogenates from mice infected with the inoculums listed in panel A. (TIF) [file ppat.1014456.s005.tif]

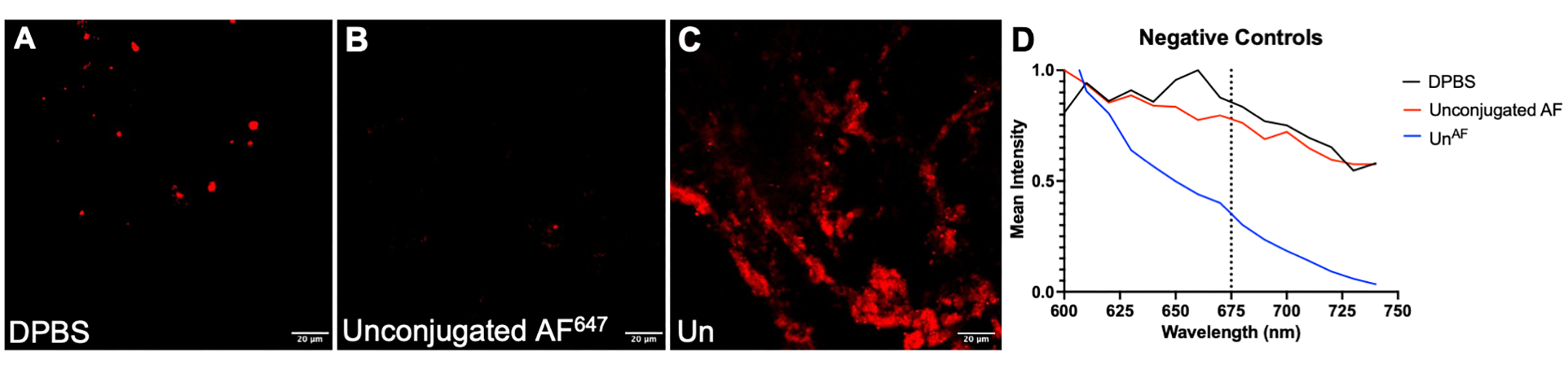

Supplement: S6 Fig — Still images from mouse sciatic nerves inoculated with either A) DPBS, B) Unconjugated AF647 quenched by glycine, or C) Uninfected purified preparations conjugated to AF647. Lambda spectral scans (D) of fluorescence from A-C are inconsistent with the AF647 spectral fingerprint. Scale bar represents 20 μm. (TIF) [file ppat.1014456.s006.tif]
