# Supplementary material for: The axonal transport velocity of prions is independent of prion formation
Source: PLoS Pathog. 2026 Jul 24;22(7):e1014456. doi: 10.1371/journal.ppat.1014456 (PMC13423175; doi:10.1371/journal.ppat.1014456)
Supplement: S1 File — (PDF) [file ppat.1014456.s009.pdf]

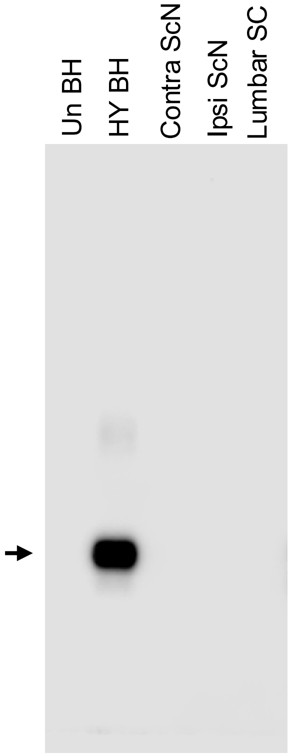

Figure 1C

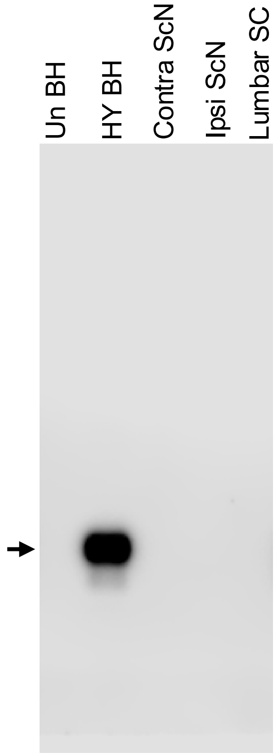

Figure 1C

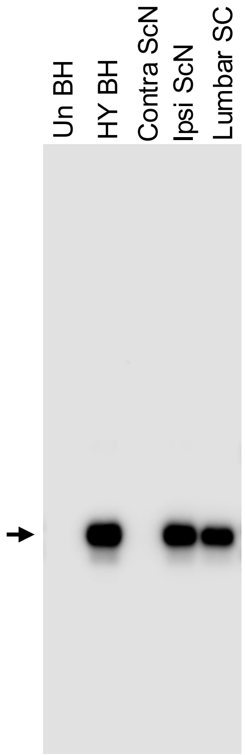

Figure 1C

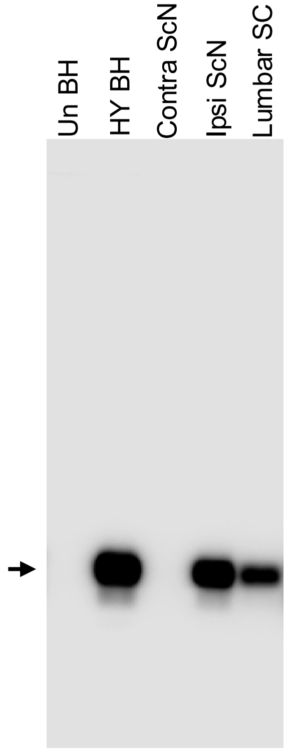

Figure 1C

- All western blots in Figure 1C were exposed with ECL reagent and imaged via chemiluminescence in the Li-Cor Odyssey XF imager (Li-Cor Biosciences, Lincoln, NE).

- Arrows indicate the migration of the 29 kDa molecular weight marker.

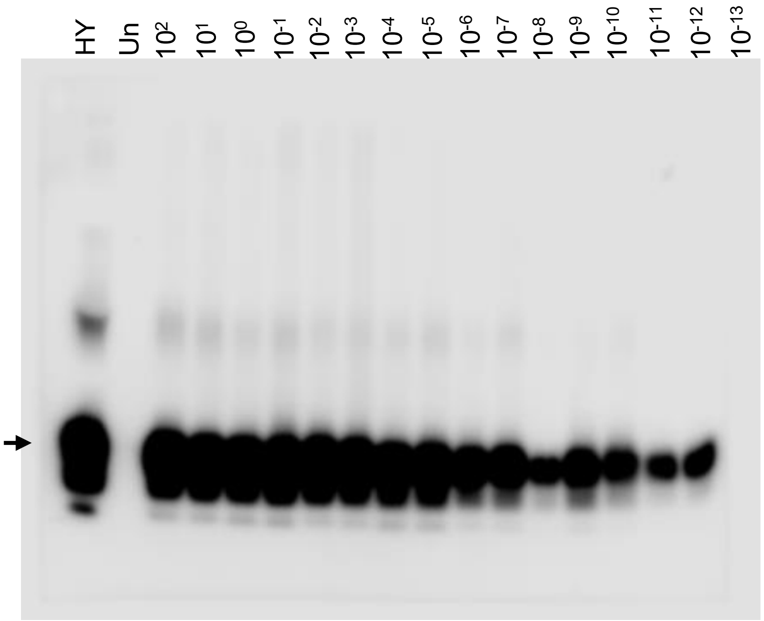

Figure S1A

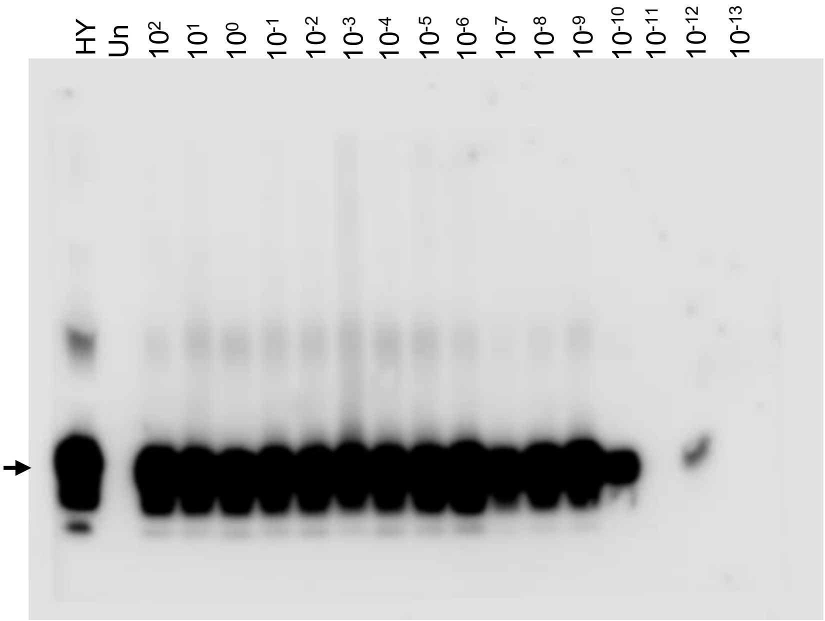

Figure S1A

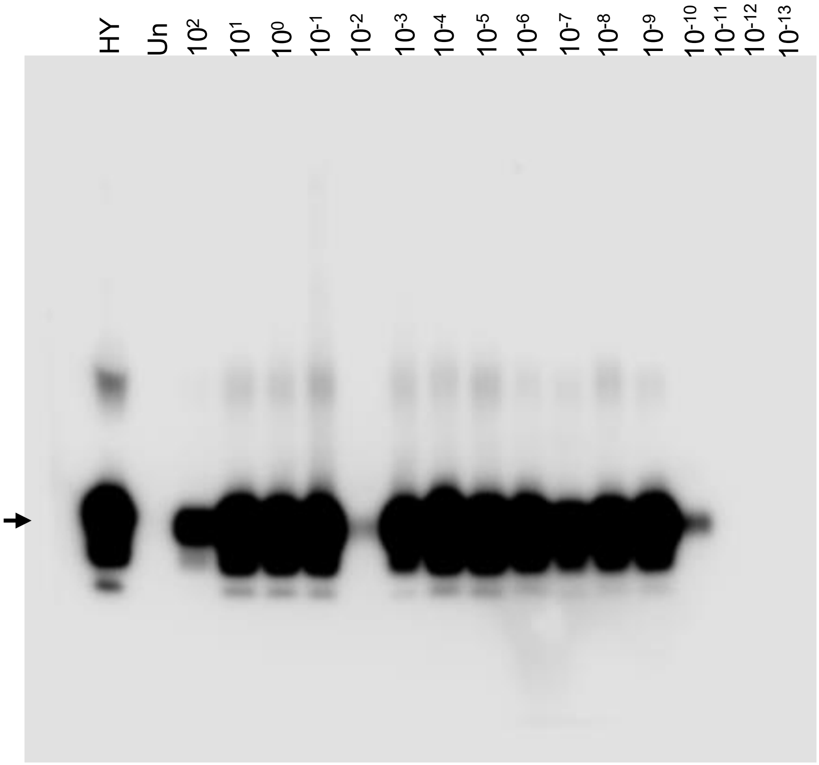

Figure S1A

- All western blots in Figure S1 Panel A were exposed with ECL reagent and imaged via chemiluminescence in the Li-Cor Odyssey XF imager (Li-Cor Biosciences, Lincoln, NE).
- Arrows indicate the migration of the 29 kDa molecular weight marker.

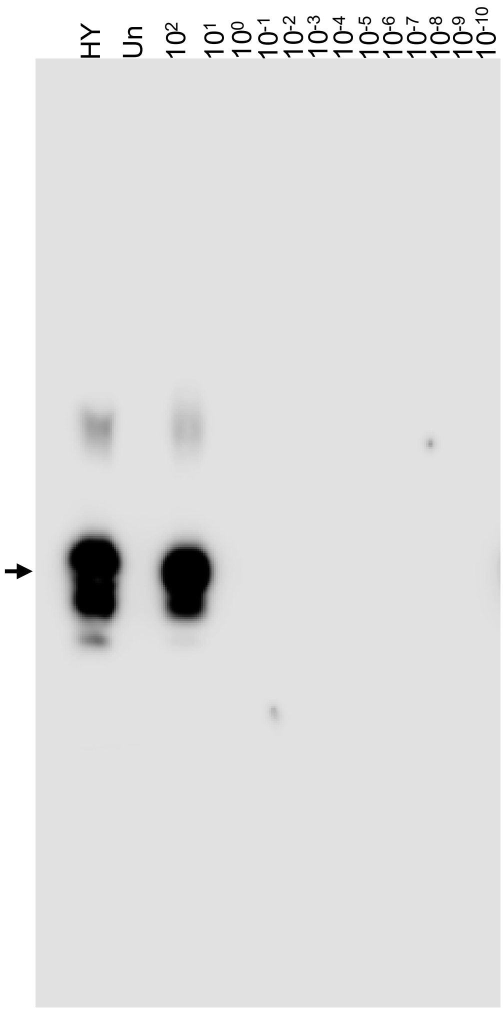

Figure S1B

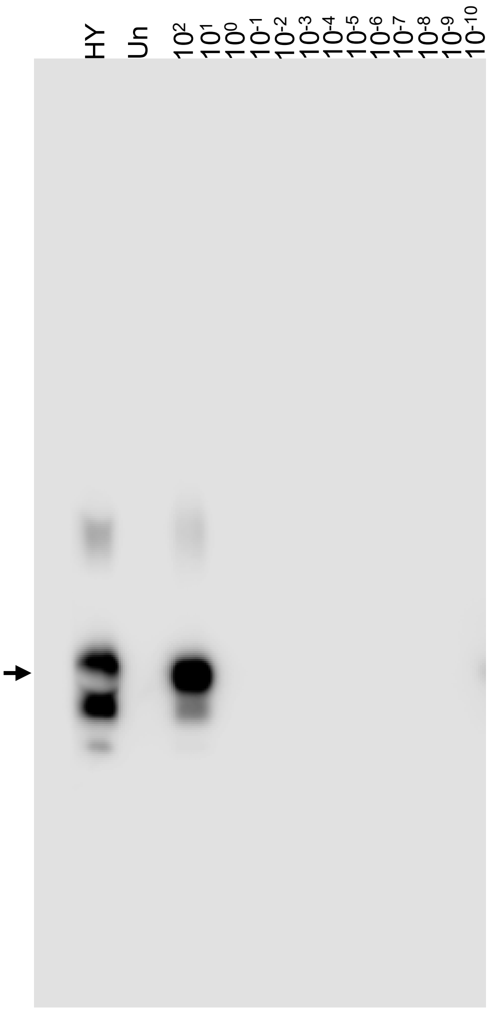

Figure S1B

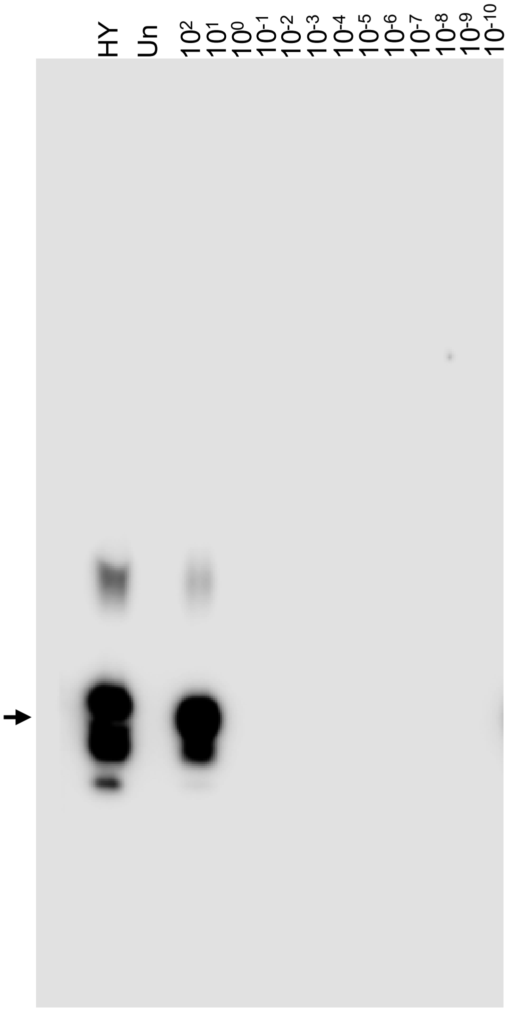

Figure S1B

- All western blots in Figure S1 Panel B. were exposed with ECL reagent and imaged via chemiluminescence in the Li-Cor Odyssey XF imager (Li-Cor Biosciences, Lincoln, NE).
- Arrows indicate the migration of the 29 kDa molecular weight marker.

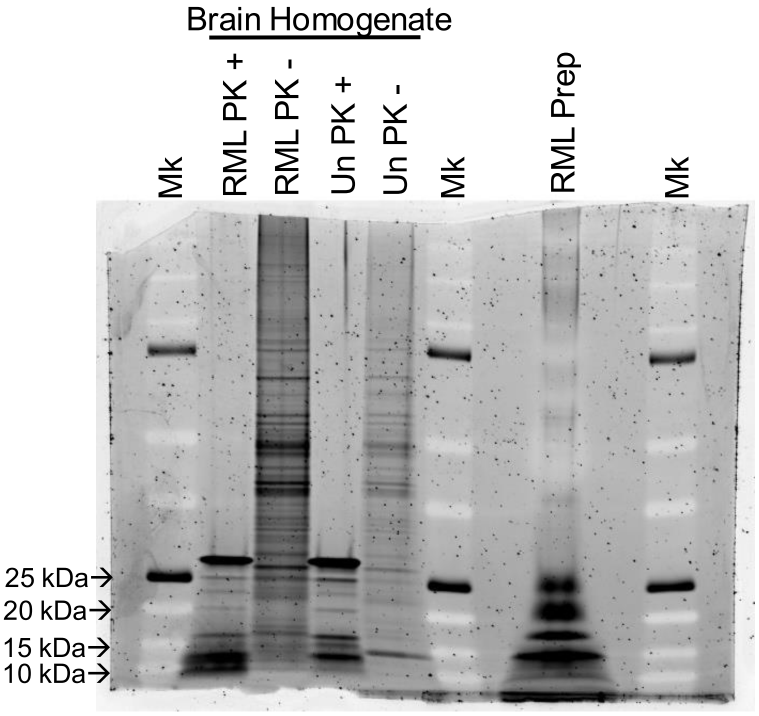

Figure S2A

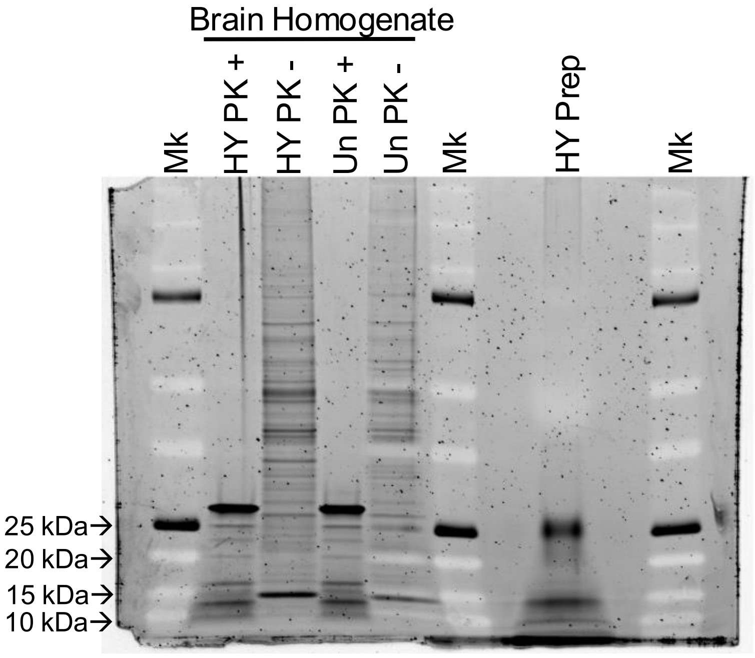

Figure S2B

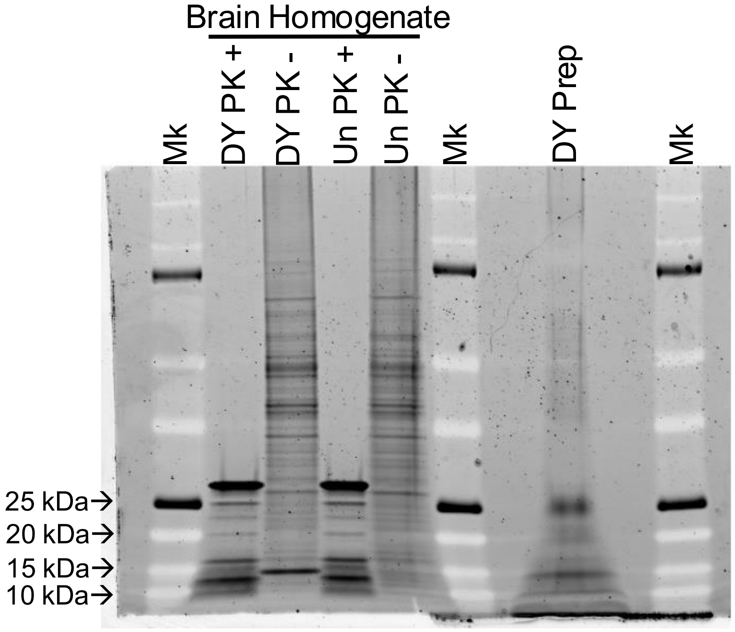

Figure S2C

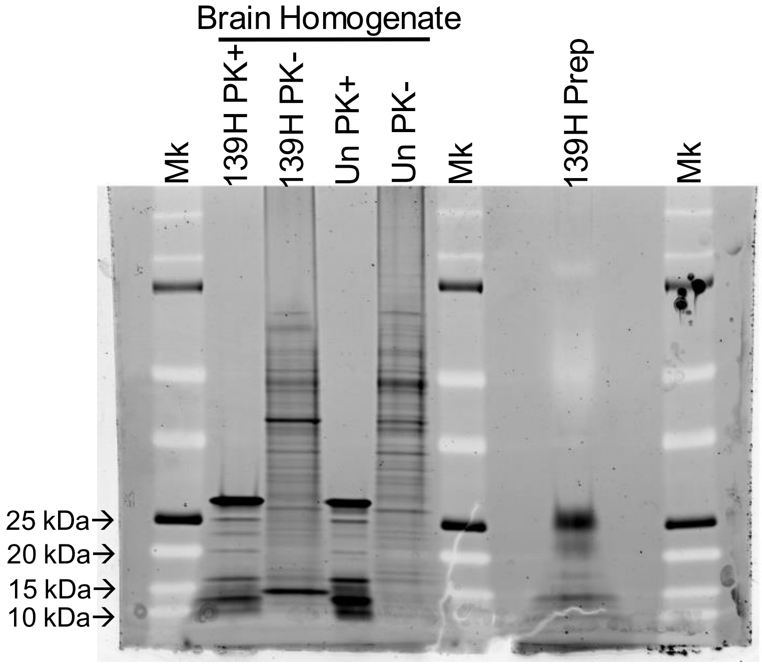

Figure S2D

- All gels in Figure S2 were imaged with the Cytivia typhoon scanner (Marlborough, MA) using the Cy3 laser and filter set (excitation: 532 nm, emission: 560-580 nm, PMT: 500-600 V).

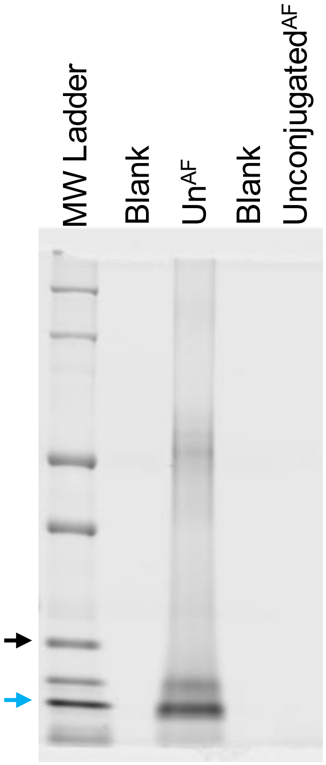

Figure S3

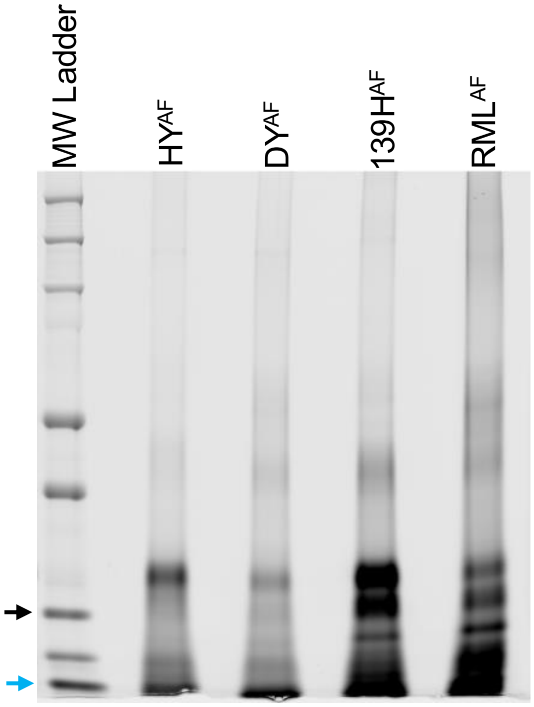

Figure S3

-For both Figure S3 gels, successful conjugation of PrP^{Sc} was confirmed by fractionating the samples on 4-12% Bis-Tris NuPage polyacrylamide gels and imaging with the Cytivia Typhoon scanner using the Cy5 laser and filter set (excitation: 635 nm, emission: 655-685 nm, PMT: 500 V).
- Black and blue arrows indicate the migration of the 20 kDa and 10 kDa molecular weight marker, respectively.

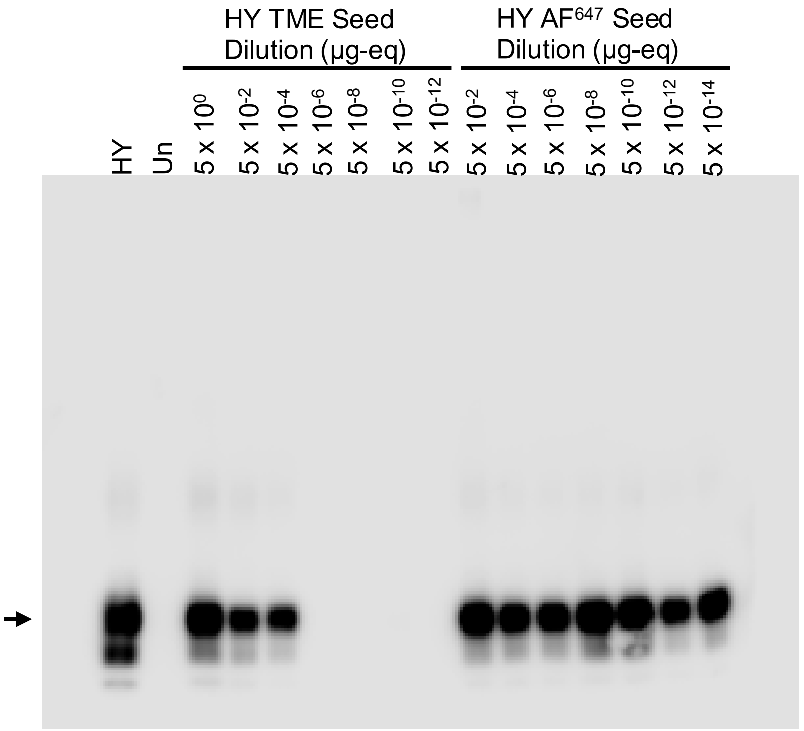

Figure S4

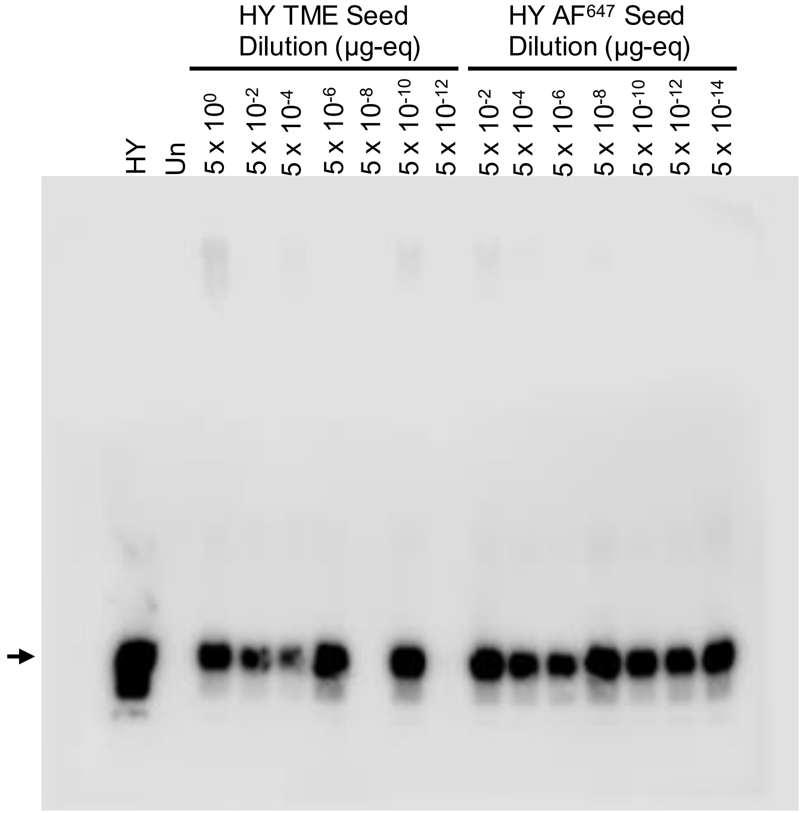

Figure S4

- All western blots in Figure S4. were exposed with ECL reagent and imaged via chemiluminescence in the Li-Cor Odyssey XF imager (Li-Cor Biosciences, Lincoln, NE).
- Arrows indicate the migration of the 29 kDa molecular weight marker.

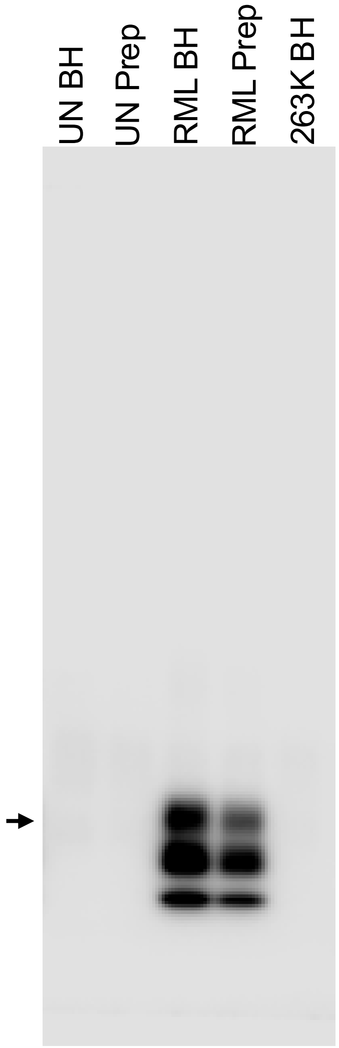

Figure S5B

- Western blot in Figure S5. Panel B was exposed with ECL reagent and imaged via chemiluminescence in the Li-Cor Odyssey XF imager (Li-Cor Biosciences, Lincoln, NE).
- Arrows indicate the migration of the 29 kDa molecular weight marker.
