# Supplementary material for: The axonal transport velocity of prions is independent of prion formation
Source: PLoS Pathog. 2026 Jul 24;22(7):e1014456. doi: 10.1371/journal.ppat.1014456 (PMC13423175; doi:10.1371/journal.ppat.1014456)
Supplement: S1 Table — (DOCX) [file ppat.1014456.s007.docx]

**Table S1.** Comparison of average axonal velocities of PrP^Sc^ strains between PrP^+/+^ and PrP^-/-^ mouse sciatic nerve explant genotypes within mouse sex.

| Prion strain | Male | Female |
| --- | --- | --- |
| RML | -0.2233±0.0666^a^  (-3.350*)^b^ | 0.0386±0.0496  (0.777) |
| HY TME | 0.1667±0.0428  (3.895*) | -0.2790±0.0279  (-10.005*) |
| DY TME | 0.2317±0.0443  (5.235*) | -0.0677±0.0507  (-1.337) |
| 139H | -0.3476±0.0338  (-10.295*) | 0.0481±0.0338  (1.420) |

^a^mean difference ± SEM

^b^T value

*p <0.05
