# Supplementary material for: The axonal transport velocity of prions is independent of prion formation
Source: PLoS Pathog. 2026 Jul 24;22(7):e1014456. doi: 10.1371/journal.ppat.1014456 (PMC13423175; doi:10.1371/journal.ppat.1014456)
Supplement: S2 Table — (DOCX) [file ppat.1014456.s008.docx]

**Table S2.** Comparison of average axonal velocities of PrP^Sc^ strains between male PrP^+/+^ and male PrP^-/-^ in live mouse sciatic nerve axons.

| Prion strain | Mean velocity difference | T value |
| --- | --- | --- |
| RML | -0.1822±0.0245^a^ | -7.441* |
| HY TME | 0.1727±0.0237 | 7.299* |
| DY TME | -0.0949±0.0261 | -3.641* |
| 139H | -0.0547±0.0256 | -2.136* |
| Mo recPrP^Sc^ | 0.0748±0.0262 | 2.860* |

^a^mean difference ± SEM

*p <0.05
